# Supplementary material for: Proctocolectomy with permanent ileostomy is associated with improved transplant-free survival in patients with PSC
Source: JHEP Rep. 2025 Dec 22;8(3):101700. doi: 10.1016/j.jhepr.2025.101700 (PMC12934274; doi:10.1016/j.jhepr.2025.101700)
Supplement: Multimedia component 1 [file mmc1.pdf]

Proctocolectomy with permanent ileostomy is associated with improved transplant-free survival in patients with PSC

Bregje Mol, Moyrha van Nieuwamerongen, Kim van Munster, Martti Färkkilä, Trine Folseraas, Sara Tjønnfjord, Johannes Hov, Kirsten Boberg, Mette Vesterhus, Kristin Jørgensen, Annika Bergquist, Jorn Goet, Annemarie de Vries, Adriaan van der Meer, Rinse Weersma, Akin Inderson, Johannes Bogaards, Cyriel Ponsioen on behalf of the IPSCR study group

Table of contents

Supplementary file……………………………………………………………………..2

Fig. S1……………………………………………………………………………….3

Table S1…………………………………………………………………………......8

Fig. S2………………………………………………………………………………12

Table S2……………………………………………………………………….....13

Table S3……………………………………………………………………….....15

Table S4……………………………………………………………………….....17

Table S5……………………………………………………………………….....19

Table S6……………………………………………………………………….....20

Supplementary references……………………………………………………..…..…...21

Supplementary File 1. Description of the separate cohorts

The Finnish cohort consists of patients retrieved from the Helsinki University Hospital (HUH) PSC-registry, Finland. HUH is a referral centre responsible for the Helsinki and Uusimaa hospital district, comprising 39% of the Finnish population. All patients referred to HUH for a diagnostic, follow-up, or surveillance ERCP were recruited from 2009 onward. Data were collected up to liver transplantation, death or January 2024.

The Dutch cohort consists of patients derived from 46 hospitals participating in the national dynamic EpiPSC2 registry. The registry was initiated in 2008 as the population based EpiPSC/PBC study and transitioned to the prospective EpiPSC2 study in 2016. All eligible participants, in compliance with GDPR, were invited to participate in the prospective arm of the study. Patients were followed up to liver transplantation, death or data lock in January 2022. Clinical data were sourced from local electronic hospital databases and written correspondence from treating physicians. All patients participating in the prospective arm of the study provided written informed consent.

The Norwegian cohort consists of patients with PSC prospectively recruited at admission to the tertiary care hospital Oslo University Hospital, Rikshospitalet (Oslo, Norway) between 2008 and 2022. Additionally, patients were selected from the prospective NoPSC national database. All non-transplanted patients with a diagnosis of PSC at baseline were included in the prospective NoPSC national database by May 2023 from Oslo University Hospital Rikshospitalet, Oslo; Akershus University Hospital, Oslo; and Harladsplass Deaconess Hospital, Bergen. Clinical follow-up data was collected up to liver transplantation, death or May 2023.

The Swedish cohort consists of patients from the Karolinska University hospital Huddinge included in the Swedish prospective surveillance study. Patients were included between November 2011 and April 2016 in a 5-year surveillance programme. Exclusion criteria were expected listing for liver transplantation within one year, previously transplanted patients and presence of a hepatobiliary malignancy. Patients were followed up to liver transplantation, death or August 2023. Clinical data were obtained by the treating physician. All patients provided written informed consent.

Fig. S1. Survival plots of all patients stratified per country for the endpoint LT or PSC-related death (excluding CRC).

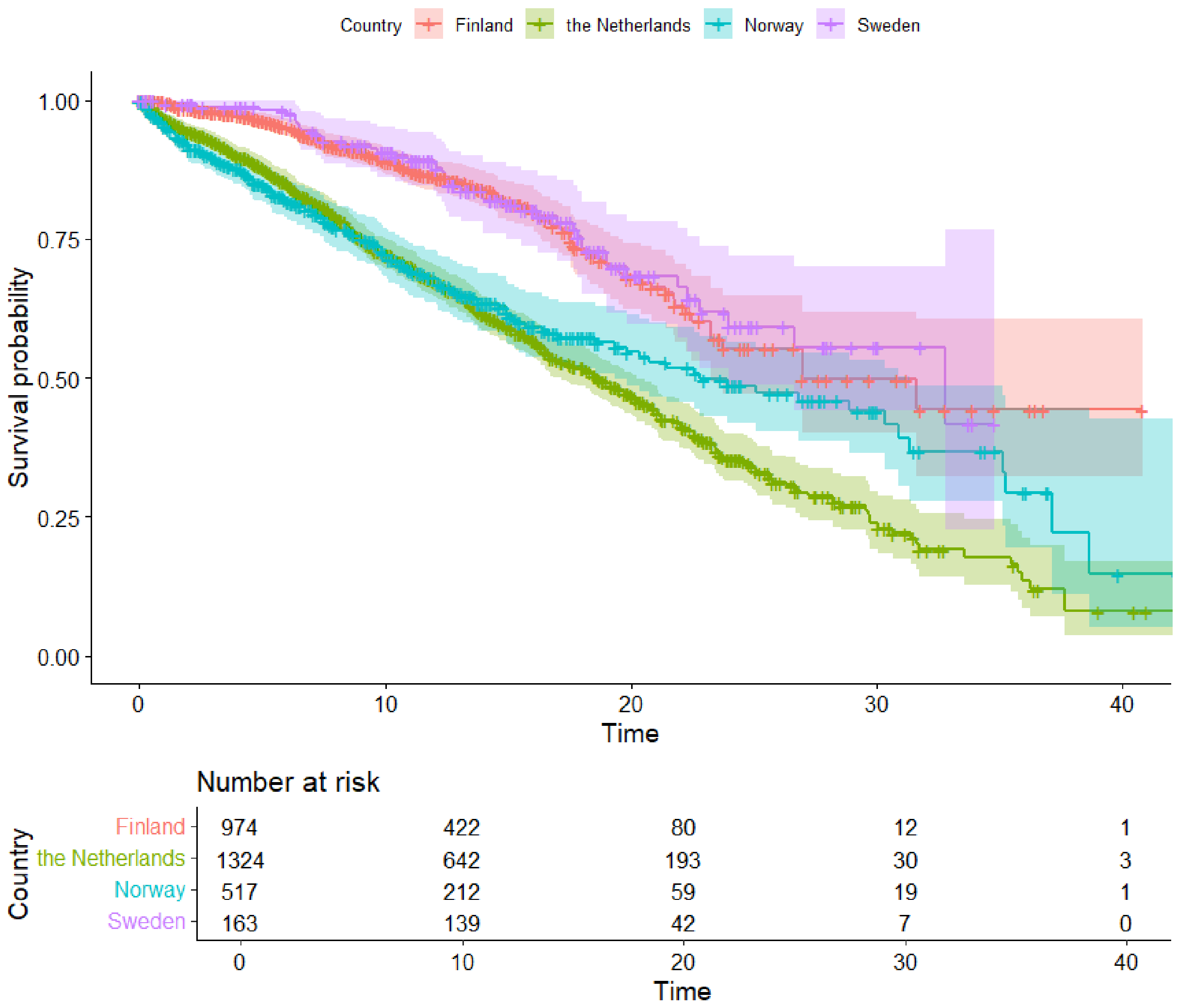

Table S1. Multivariable model results for transplant-free survival defined as time up to liver transplantation of PSC-related death (excluding colorectal carcinoma) stratified per country

A. Finland		HR (95%CI)
Colectomy	*None*	Reference
	Subtotal colectomy or hemicolectomy	0.90 (0.12-6.57)
	Proctocolectomy with pouch	0.96 (0.44-2.11)
	Proctocolectomy with ileostomy	0.98 (0.13-7.27)
Female		0.89 (0.62-1.27)
Age at diagnosis (per year)		1.02 (1.01-1.04)
PSC type	*Large duct*	Reference
	Small duct	N.A.
	Features of AIH	2.08 (1.30-3.33)
IBD	*No IBD*	Reference
	Ulcerative colitis	1.08 (0.69-1.69)
	Crohn's disease	0.86 (0.48-1.56)
	IBD-unspecified	1.76 (0.61-5.09)
Transplant centre patient		0.60 (0.42-0.86)
B. The Netherlands		HR (95%CI)
Colectomy	*None*	Reference
	Subtotal colectomy or hemicolectomy	0.82 (0.56-1.20)

	Proctocolectomy with pouch<br>*Proctocolectomy with ileostomy*	0.99 (0.65-1.52)<br>0.43 (0.21-0.90)
Female		0.82 (0.68-0.99)
Age at diagnosis (per year)		1.02 (1.01-1.02)
PSC type	*Large duct*<br>*Small duct*<br>*Features of AIH*	Reference<br>0.23 (0.12-0.48)<br>0.83 (0.60-1.15)
IBD	*No IBD*<br>*Ulcerative colitis*<br>*Crohn's disease*<br>*IBD-unspecified*	Reference<br>1.04 (0.84-1.28)<br>0.88 (0.65-1.18)<br>0.76 (0.44-1.33)
Transplant centre patient		1.90 (1.60-2.26)
C. Norway		HR (95%CI)
Colectomy	*None*<br>Subtotal colectomy or hemicolectomy<br>*Proctocolectomy with pouch*<br>*Proctocolectomy with ileostomy*	Reference<br>0.56 (0.24-1.28)<br>0.77 (0.39-1.52)<br>0.33 (0.13-0.86)
Female		0.80 (0.56-1.16)
Age at diagnosis (per year)		1.04 (1.03-1.05)

PSC type	*Large duct*<br>*Small duct*<br>*Features of AIH*	Reference<br>0.00 (0.00-N.A.)<br>1.25 (0.67-2.35)
IBD	*No IBD*<br>*Ulcerative colitis*<br>*Crohn's disease*<br>*IBD-unspecified*	Reference<br>0.90 (0.61-1.33)<br>0.75 (0.40-1.40)<br>1.59 (0.70-3.63)
Transplant centre patient		5.61 (3.51-8.96)
D. Sweden		HR (95%CI)
Colectomy	*None*<br>Subtotal colectomy or hemicolectomy<br>*Proctocolectomy with pouch*<br>*Proctocolectomy with ileostomy*	Reference<br>2.92 (0.67-12.66)<br>0.40 (0.09-1.81)<br>0.00 (0.00-N.A.)
Female		0.89 (0.46-1.75)
Age at diagnosis (per year)		1.01 (0.99-1.04)
PSC type	*Large duct*<br>*Small duct*<br>*Features of AIH*	Reference<br>0.00 (0.00-N.A.)<br>1.06 (0.44-2.56)
IBD	*No IBD*<br>*Ulcerative colitis*	Reference<br>0.77 (0.37-1.60)

	Crohn's disease<br>*IBD-unspecified*	0.41 (0.13-1.33)<br>1.00 (0.13-7.96)
Transplant centre patient		N.A.

Fig. S2A-D. Predicted survival probabilities for a 37 year-old male with large duct PSC and ulcerative colitis from the Netherlands (A), Finland (B), Norway (C), Sweden (D) for the endpoint LT or PSC-related death (excluding CRC).

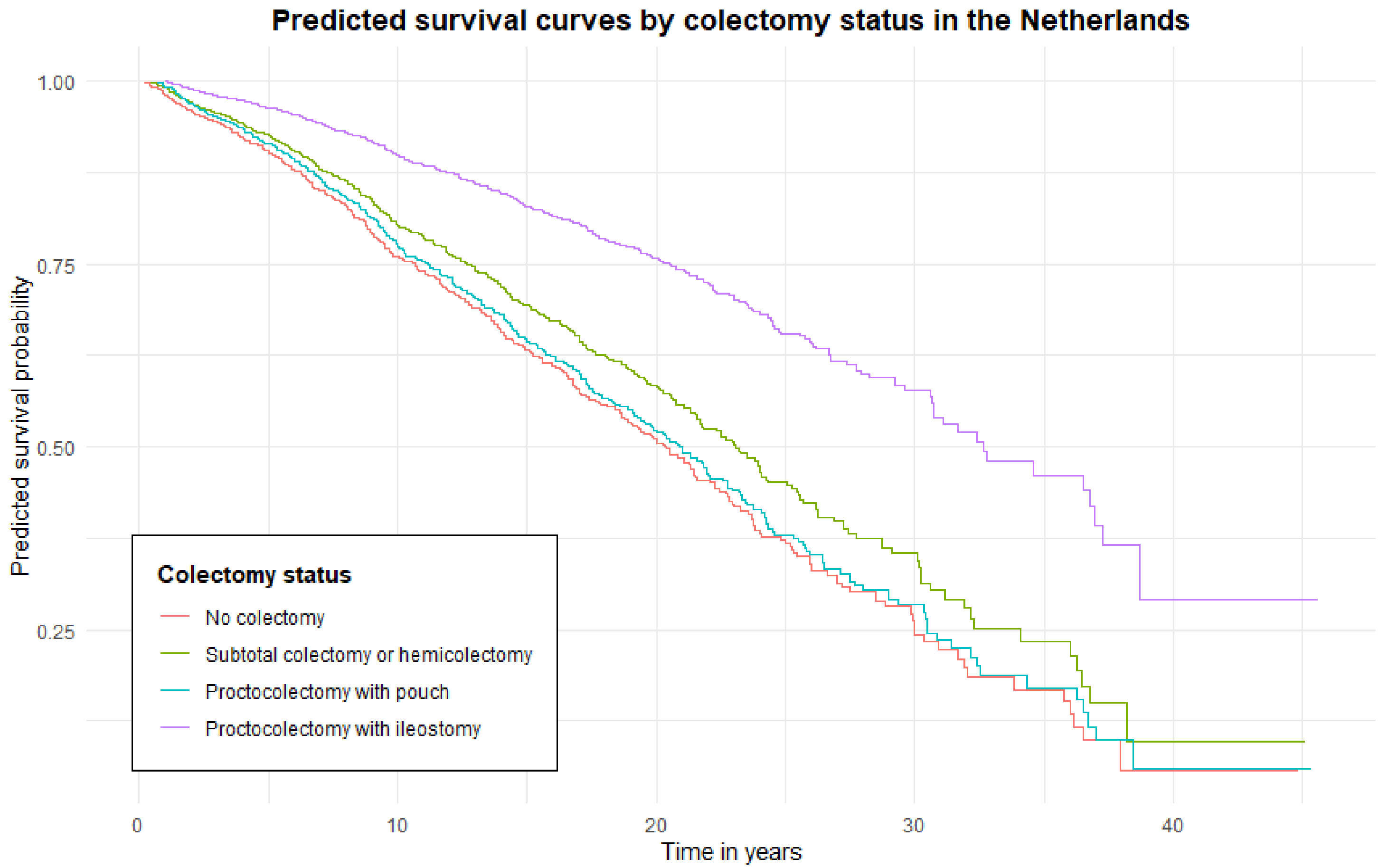

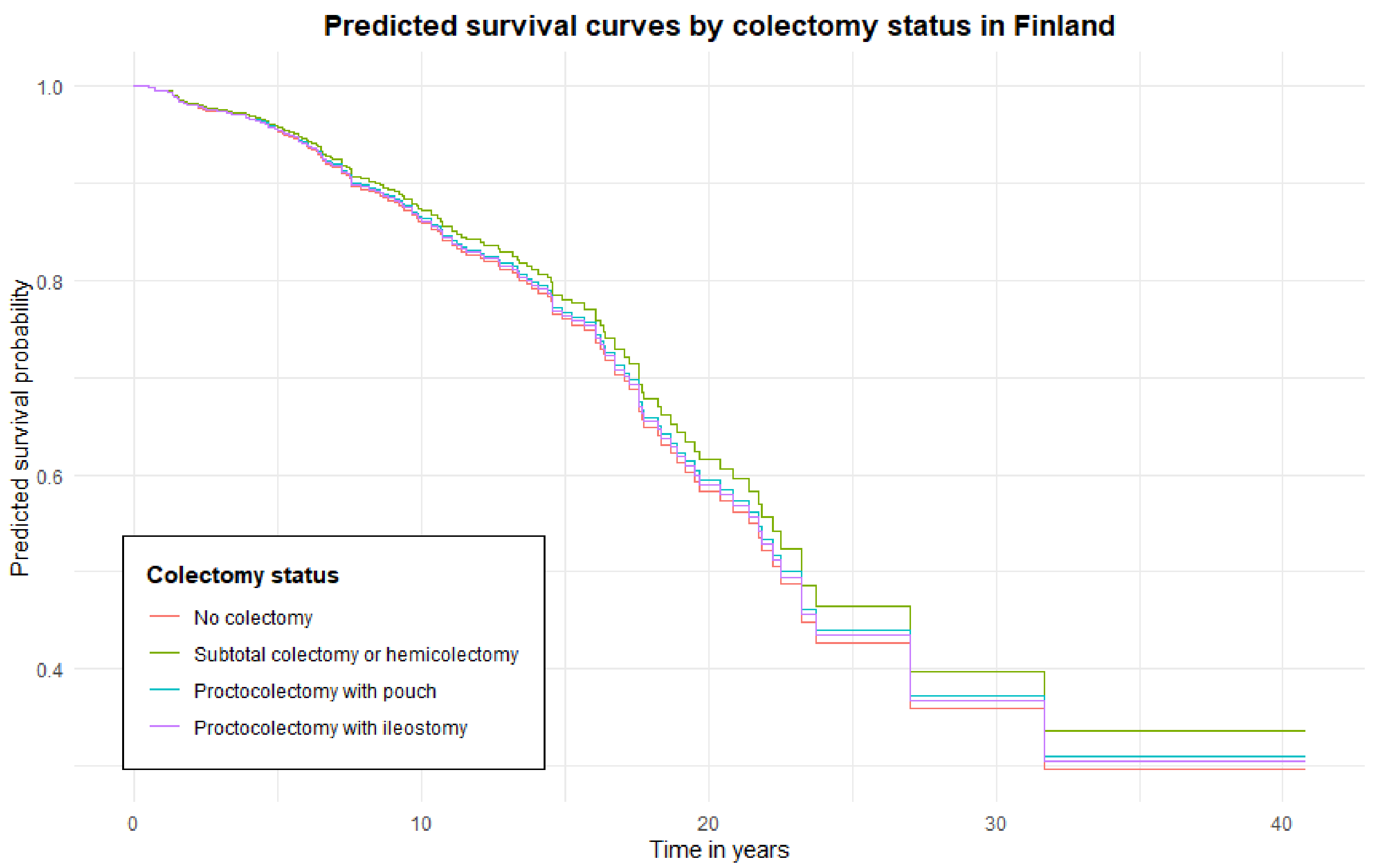

Predicted survival curves by colectomy status in Finland
Predicted survival probability
1.0
0.8
0.6
0.4
0
10
20
30
40
Time in years
Colectomy status
No colectomy
Subtotal colectomy or hemicolectomy
Proctocolectomy with pouch
Proctocolectomy with ileostomy

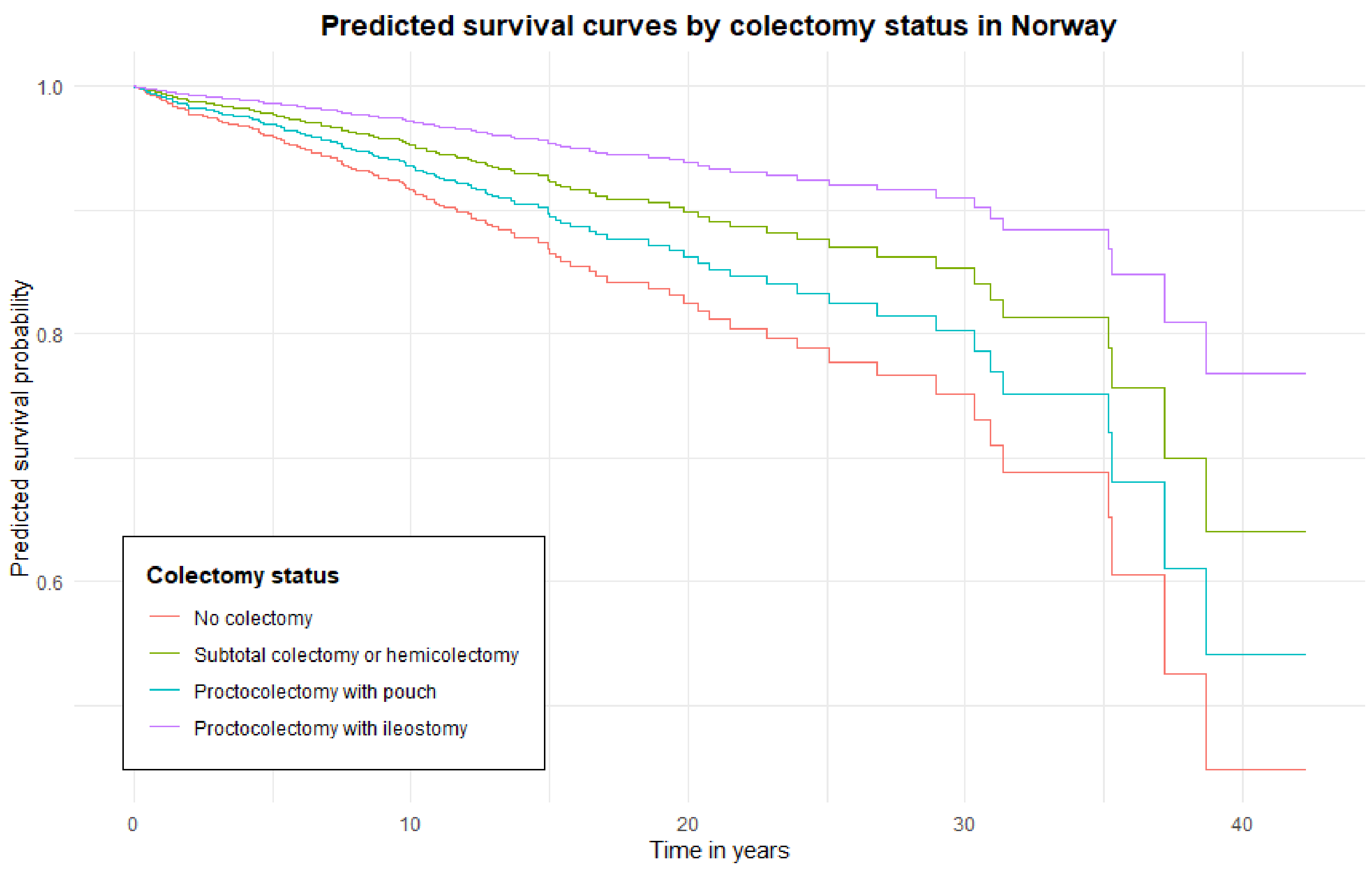
Predicted survival curves by colectomy status in Norway
1.0
0.8
0.6
Predicted survival probability
0
10
20
30
40
Time in years
Colectomy status
No colectomy
Subtotal colectomy or hemicolectomy
Proctocolectomy with pouch
Proctocolectomy with ileostomy

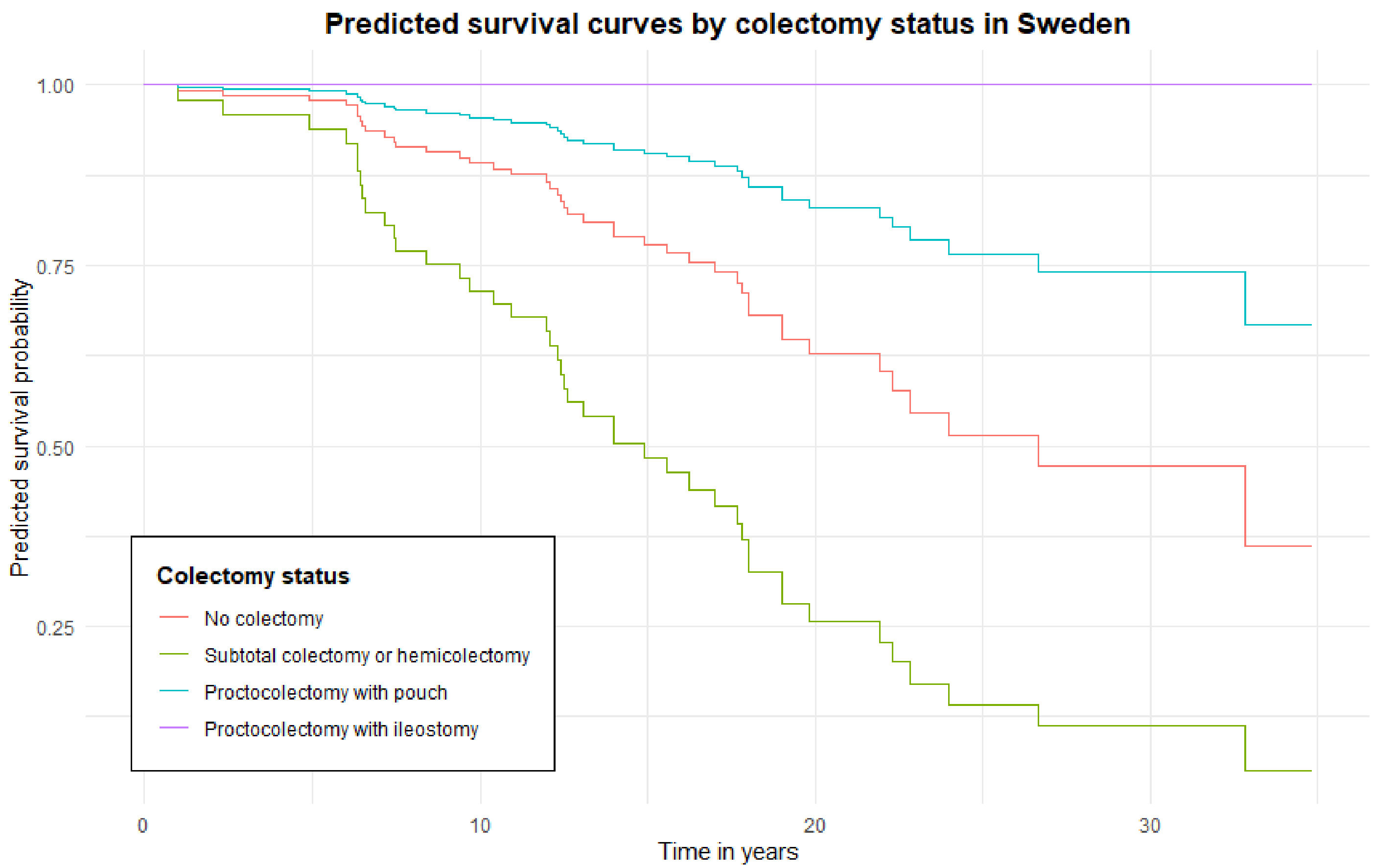

Predicted survival curves by colectomy status in Sweden
1.00
0.75
0.50
0.25
Predicted survival probability
0
10
20
30
Time in years
Colectomy status
No colectomy
Subtotal colectomy or hemicolectomy
Proctocolectomy with pouch
Proctocolectomy with ileostomy

Table S2. Multivariable model results for the sensitivity analysis with assigning events within the first year after colectomy to the pre-colectomy period (A) and without censoring events within one year after colectomy (B)

A. Events within the first year after colectomy assigned to the pre-colectomy period		HR (95%CI)
Colectomy	*None*<br>Subtotal colectomy or<br>hemicolectomy*Proctocolectomy with*<br>*pouch*<br>*Proctocolectomy with ileostomy*	Reference<br>0.91 (0.67-1.25)<br>1.09 (0.81-1.48)<br>0.47 (0.28-0.79)
Female		0.79 (0.69-0.92)
Age at diagnosis (per year)		1.02 (1.02-1.03)
PSC type	*Large duct*<br>*Small duct*<br>*Features of AIH*	Reference<br>0.22 (0.11-0.45)<br>1.06 (0.84-1.34)
IBD	*No IBD*<br>*Ulcerative colitis*<br>*Crohn's disease*<br>*IBD-unspecified*	Reference<br>1.02 (0.86-1.20)<br>0.80 (0.63-1.01)<br>0.95 (0.63-1.43)
Country	*the Netherlands*<br>*Finland*	Reference<br>0.58 (0.42-0.81)

	Norway<br>*Sweden*	0.78 (0.66-0.94)<br>0.36 (0.30-0.44)
Transplant centre patient		1.82 (1.58-2.09)
B. Without censoring events within one year after colectomy		HR (95%CI)
Colectomy	*None*<br>Subtotal colectomy or hemicolectomy<br>*Proctocolectomy with pouch*<br>*Proctocolectomy with ileostomy*	Reference<br>0.94 (0.69-1.27)<br>1.09 (0.81-1.49)<br>0.50 (0.30-0.83)
Female		0.79 (0.68-0.92)
Age at diagnosis (per year)		1.02 (1.02-1.03)
PSC type	*Large duct*<br>*Small duct*<br>*Features of AIH*	Reference<br>0.22 (0.11-0.45)<br>1.06 (0.84-1.34)
IBD	*No IBD*<br>*Ulcerative colitis*<br>*Crohn's disease*<br>*IBD-unspecified*	Reference<br>1.02 (0.86-1.20)<br>0.80 (0.63-1.02)<br>0.95 (0.63-1.43)
Country	*the Netherlands*<br>*Finland*	Reference<br>0.58 (0.42-0.80)

	Norway<br>*Sweden*	0.78 (0.65-0.93)<br>0.36 (0.30-0.44)
Transplant centre patient		1.81 (1.58-2.09)

Table S3. Multivariable model results for transplant-free survival defined as time up to liver transplantation of PSC-related death (excluding colorectal carcinoma) for only participants with a diagnosis of both PSC and IBD.

A. Liver transplantation (excluding HB malignancy) or death due to liver failure		HR (95%CI)
Colectomy	*None*	Reference
	Subtotal colectomy or hemicolectomy	0.76 (0.53-1.09)
	Proctocolectomy with pouch	0.55 (0.24-1.27)
	Proctocolectomy with ileostomy	0.24 (0.09-0.61)
Female		0.85 (0.71-1.01)
Age at diagnosis (per year)		1.02 (1.02-1.03)
PSC type	*Large duct*	Reference
	Small duct	0.26 (0.10-0.69)
	Features of AIH	1.04 (0.78-1.38)
IBD	*No IBD*	Reference
	Ulcerative colitis	0.56 (0.26-1.24)
	Crohn's disease	0.44 (0.20-0.99)
	IBD-unspecified	0.52 (0.22-1.24)
Country	*the Netherlands*	Reference
	Finland	0.50 (0.35-0.73)
	Norway	0.75 (0.61-0.92)
	Sweden	0.38 (0.30-0.47)

Transplant centre patient		1.64 (1.40-1.93)

Table S4. Multivariable model results for the secondary endpoints liver transplantation (excluding hepatobiliary malignancy) or death due to liver failure (A) and occurrence of hepatobiliary malignancy (B)

A. Liver transplantation (excluding HB malignancy) or death due to liver failure		HR (95%CI)
Colectomy	*None*	Reference
	Subtotal colectomy or hemicolectomy	0.65 (0.43-0.97)
	Proctocolectomy with pouch	0.75 (0.50-1.11)
	Proctocolectomy with ileostomy	0.24 (0.10-0.53)
Female		0.81 (0.69-0.96)
Age at diagnosis (per year)		1.02 (1.01-1.02)
PSC type	*Large duct*	Reference
	Small duct	0.18 (0.08-0.45)
	Features of AIH	1.03 (0.80-1.34)
IBD	*No IBD*	Reference
	Ulcerative colitis	1.07 (0.89-1.29)
	Crohn's disease	0.78 (0.60-1.02)
	IBD-unspecified	0.98 (0.62-1.67)
Country	*the Netherlands*	Reference
	Finland	0.71 (0.51-1.00)
	Norway	0.74 (0.60-0.91)
	Sweden	0.33 (0.2-0.41)

Transplant centre patient		2.00 (1.71-2.36)
B. Occurrence of HB malignancy		HR (95%CI)
Colectomy	*None*	Reference
	Subtotal colectomy or hemicolectomy	1.26 (0.75-2.13)
	Proctocolectomy with pouch	1.62 (0.96-2.75)
	Proctocolectomy with ileostomy	1.17 (0.60-2.29)
Female		0.74 (0.56-1.00)
Age at diagnosis (per year)		1.04 (1.03-1.05)
PSC type	*Large duct*	Reference
	Small duct	0.48 (0.18-1.31)
	Features of AIH	0.96 (0.57-1.63)
IBD	*No IBD*	Reference
	Ulcerative colitis	1.01 (0.73-1.42)
	Crohn's disease	0.91 (0.57-1.47)
	IBD-unspecified	1.22 (0.57-2.59)
Country	*the Netherlands*	Reference
	Finland	0.00 (0.00-N.A.)
	Norway	1.00 (0.73-1.38)
	Sweden	1.11 (0.74-1.69)

Transplant centre patient		2.02 (1.51-2.70)

Table S5. Colectomy indication stratified by colectomy type

	Hemicolectomy (N=39)	Subtotal colectomy (N=93)	Proctocolectomy with ileal-anal pouch anastomosis (N=232)	Proctocolectomy with end-ileostomy (N=80)
Active disease	11 (28%)	48 (52%)	116 (50%)	48 (50%)
Active disease and neoplasia	0 (0%)	2 (2%)	9 (4%)	1 (1%)
Dysplasia or carcinoma	21 (54%)	38 (41%)	79 (34%)	29 (36%)
Other	6 (15%)	2 (2%)	3 (1%)	0 (0%)
Unknown	1 (3%)	3 (3%)	25 (11%)	8 (10%)
Colectomy indication stratified by colectomy type.				

Table S6. Multivariable model results for indication of colectomy for the endpoint liver transplantation or PSC-related death (excluding colorectal carcinoma) including only time at risk after any type of colectomy

A. Liver transplantation or PSC-related death (excluding colorectal carcinoma)		HR (95%CI)
Colectomy indication	*Active disease*	Reference
	Active disease and neoplasia	1.42 (0.49-4.11)
	Dysplasia or carcinoma	0.74 (0.46-1.19)
	Other	1.77 (0.68-4.64)
	Unknown	1.10 (0.54-2.26)
Colectomy	*None*	N.A.
	Subtotal colectomy or hemicolectomy	2.07 (0.77-5.54)
	Proctocolectomy with pouch	2.01 (1.04-3.88)
	Proctocolectomy with ileostomy	Reference
Female		1.27 (0.81-2.01)
Age at diagnosis (per year)		1.01 (0.99-1.02)
PSC type	*Large duct*	Reference
	Small duct	0.32 (0.04-2.38)
	Features of AIH	1.44 (0.68-3.06)
IBD	*No IBD*	Reference
	Ulcerative colitis	0.75 (0.30-1.87)

	Crohn's disease<br>*IBD-unspecified*	0.50 (0.16-1.59)<br>0.56 (0.11-2.81)
Country	*the Netherlands*<br>*Finland*<br>*Norway*<br>*Sweden*	Reference<br>0.39 (0.13-1.13)<br>0.57 (0.34-0.96)<br>0.56 (0.27-1.17)
Transplant centre patient		1.51 (0.95-2.40)

Supplementary references

1. van Munster KN, Mol B, Goet JC, et al. Disease burden in primary sclerosing cholangitis in the Netherlands: A long-term follow-up study. *Liver Int* 2023; **43**(3): 639-48.
2. Boonstra K, Weersma RK, van Erpecum KJ, et al. Population-based epidemiology, malignancy risk, and outcome of primary sclerosing cholangitis. *Hepatology* 2013; **58**(6): 2045-55.
3. Villard C, Friis-Liby I, Rorsman F, et al. Prospective surveillance for cholangiocarcinoma in unselected individuals with primary sclerosing cholangitis. *J Hepatol* 2023; **78**(3): 604-13.
