# Supplementary material for: Proctocolectomy with permanent ileostomy is associated with improved transplant-free survival in patients with PSC
Source: JHEP Rep. 2025 Dec 22;8(3):101700. doi: 10.1016/j.jhepr.2025.101700 (PMC12934274; doi:10.1016/j.jhepr.2025.101700)
Supplement: Multimedia component 4 [file mmc4.pdf]

JHEP Reports

Proctocolectomy with permanent ileostomy is associated with improved transplant-free survival in patients with PSC

Authors

Bregje Mol, Moyrha van Nieuwamerongen, Kim N. van Munster, …, Akin Inderson, Johannes A. Bogaards, Cyriel Y. Ponsioen

Correspondence

c.y.ponsioen@amsterdamumc.nl (C.Y. Ponsioen).

Graphical abstract

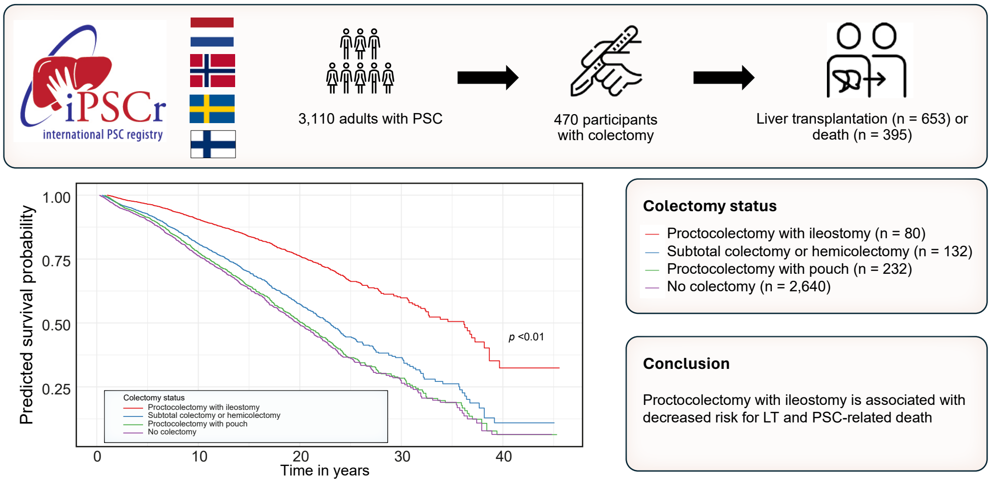

Highlights:

- Proctocolectomy with ileostomy was linked to fewer LTs and deaths.
- Hemi- or subtotal colectomy offered only a small protective effect.
- No benefit was seen after proctocolectomy with pouch.
- The beneficial effect of colectomy was driven by reduced risk of liver failure.

Impact and implications:

The impact of the gut-liver axis in the pathophysiology of primary sclerosing cholangitis (PSC) has remained uncertain. In this study, proctocolectomy with ileostomy was associated with improved transplant-free survival, defined as a reduced risk of liver transplantation or PSC-related death, indicating that intestinal factors may influence disease progression. These findings are important for clinicians, researchers, and patients as they suggest that surgical management of colonic disease may have prognostic implications in PSC, and for further studies to clarify mechanisms and guide clinical decision-making.

https://doi.org/10.1016/j.jhepr.2025.101700
© 2025 The Author(s). Published by Elsevier B.V. on behalf of European Association for the Study of the Liver (EASL). This is an open access article under the CC BY license (http://creativecommons.org/licenses/by/4.0/).

JHEP|Reports

Proctocolectomy with permanent ileostomy is associated with improved transplant-free survival in patients with PSC

Bregje Mol[1,2], Moyrha van Nieuwamerongen[1], Kim N. van Munster[1,2], Martti Färkkilä[3], Trine Folseraas[4,5,6,7], Sara K.V. Tjønnfjord[4,5,6], Johannes R. Hov[4,5,6,7], Kirsten Boberg[4,5,6,7], Mette Vesterhus[4,8], Kristin K. Jørgensen[4,5,9], Annika Bergquist[10], Jorn C. Goet[11], Annemarie C. de Vries[11], Adriaan J.P. van der Meer[11], Rinse K. Weersma[12], Akin Inderson[13], Johannes A. Bogaards[14,15], Cyriel Y. Ponsioen[1,2,*], on behalf of the IPSCR study group

JHEP Reports **2026**. vol. 8 | 1–8

Check for updates

Background & Aims: The gut–liver axis is believed to be crucial in the pathogenesis of primary sclerosing cholangitis (PSC). However, the impact of colectomy on liver disease progression is unclear. Our study estimated the effect of colectomy on PSC progression with correction for time dependency and established risk factors by pooling data from several cohorts across different countries.

Methods: We analysed data from the International PSC Registry (IPSCR), comprising patients from Finland, The Netherlands, Norway, and Sweden. Primary endpoint was defined as liver transplantation (LT) or PSC-related death. Cox proportional hazards regression onto time-dependent colectomy status, with specification for extent, was performed with adjustment for sex, age at diagnosis, large or small duct PSC, features of autoimmune hepatitis, time-dependent inflammatory bowel disease (IBD) status, centre of inclusion, and country of residence.

Results: A total of 3,110 participants were included, of whom 470 (15%) had undergone colectomy. During a total follow-up of 32,236 patient-years, 395 deaths and 653 LTs were observed. Compared with patients with PSC with intact colon, the hazard ratio (HR) of reaching LT or PSC-related death was significantly decreased in patients with proctocolectomy with permanent ileostomy (HR 0.41; 95% CI 0.24–0.71). This effect was less pronounced in case of hemi- or subtotal colectomy (HR 0.81; 95% CI: 0.58–1.12) and not observed for proctocolectomy with pouch (HR 1.00; 95% CI: 0.73–1.38). The reduced risk was mainly associated with a lower rate of LT or death resulting from liver failure (HR 0.24; 0.10–0.53).

Conclusions: Proctocolectomy with permanent ileostomy was associated with decreased risk for LT and PSC-related death. These findings support the role of the gut–liver axis in the pathophysiology of PSC and call for consideration in counselling patients who face impending colorectal surgery.

© 2025 The Author(s). Published by Elsevier B.V. on behalf of European Association for the Study of the Liver (EASL). This is an open access article under the CC BY license (http://creativecommons.org/licenses/by/4.0/).

Introduction

Primary sclerosing cholangitis (PSC) is a chronic cholestatic liver disease characterised by inflammation and fibrosis of the bile ducts. The aetiology of PSC remains elusive, but its strong association with inflammatory bowel disease (IBD) suggests a significant interplay between the liver and the gut, in particular the colon.[1] Currently, several hypotheses highlight the gut–liver axis as a crucial component in the pathophysiology of PSC, ranging from a leaky gut, aberrant gut-homing lymphocyte trafficking to colonic microbiota dysbiosis, or a combination thereof.[2–4]

Given the lack of a full understanding of the disease pathogenesis and the absence of targeted therapies, the therapeutic landscape for PSC remains challenging. In this context, colectomy emerges as a potential modulator of disease progression by interfering with the gut–liver axis. Colectomy in patients with PSC is not uncommon either to manage refractory IBD or as surgical treatment for high-grade dysplasia and colorectal carcinoma (CRC), a condition to which patients with PSC-IBD are particularly at risk.[5] Limited evidence suggests more favourable outcomes after liver transplantation (LT) for patients who underwent colectomy.[6] Moreover, patients who have undergone proctocolectomy with permanent ileostomy appear to have the most favourable graft outcomes.[7] Limited evidence suggests a beneficial effect of colectomy on the disease course of PSC before, as well as a more favourable outcome after, LT.[6,8] A meta-analysis by Ong *et al.*[8] combined the limited available literature and failed to demonstrate an effect of colectomy on transplant-free survival. However, the existing evidence is fraught with limitations because these studies either depend on small sample sizes, may be confounded by unobserved risk factors, or did not account for time dependency in the effect of colectomy. Time dependency implies a change in risk over time, such as in patients before

* Corresponding author. Address: Amsterdam University Medical Centers, Amsterdam, The Netherlands.
E-mail address: c.y.ponsioen@amsterdamumc.nl (C.Y. Ponsioen).
https://doi.org/10.1016/j.jhepr.2025.101700

and after colectomy. The aim of our study was to estimate the effect of colectomy on the disease course of PSC by taking time dependency and established risk factors, such as sex, age, PSC type, and diagnosis of IBD, into account. To increase statistical power and enable comparisons across settings, we set up a collaborative international cohort study for this purpose.

Patients and methods

Study design and participants

This cohort study was based on the International PSC Registry (IPSCR), an international collaboration in which all participating parties maintain a uniform registry with a common core dataset. Data from Finland, The Netherlands, Norway, and Sweden were initially collected retrospectively and are updated prospectively on an annual basis from June 2023 onward. Cohort descriptions are provided in the Supplementary Files. All participants aged 18 years or older with a diagnosis of PSC according to EASL criteria were eligible.[9]

The study was approved by the institutional review boards of the UMC Utrecht (NL14614.041.06/METC06-267/E), Helsinki University Hospital (29 §/02.02.2024;dnro HUS/1566/2020), Oslo University Hospital (2015/2140), Haraldsplass Deaconess Hospital (2018/1425/REK vest), and Karolinska Institutet (Dnr 2020-01538).

Procedures and outcomes

Data regarding colectomy status were collected retrospectively at inclusion and updated prospectively by medical chart review. The date, indication, and extent of the colectomy was registered for each surgical intervention. Indication was defined as refractory IBD with or without neoplasia; dysplasia or carcinoma; other; or unestablished. Extent of colectomy was recorded as hemicolectomy; subtotal colectomy; or proctocolectomy. In case of a proctocolectomy, whether an ileal pouch-anal anastomosis or permanent ileostomy was constructed was recorded.

The primary outcome measure was transplant-free survival, defined as time from PSC diagnosis up to the last follow-up date, LT, or PSC-related death (death resulting from liver failure; hepatobiliary cancer; or other related causes, but excluding death resulting from CRC), with observation time censored at the last follow-up date, or other causes or death. CRC was excluded from the composite endpoints to avoid confounding by indication. In addition, transplant-free survival time was calculated up to LT or all-cause mortality. At inclusion, the date of PSC diagnosis was registered. Date and cause of death were registered retrospectively if applicable and prospectively updated every year. Date and indication for LT were registered retrospectively and prospectively updated every year. Indication for transplantation was defined as end-stage liver disease; recurrent cholangitis; symptoms, including intractable itch and fatigue; cholangiocarcinoma (CCA); hepatocellular carcinoma (HCC); biliary dysplasia; or unestablished.

To distinguish between outcomes driven by liver disease progression *vs.* occurrence of hepatobiliary cancer, two secondary endpoints were constructed. The first was defined as time from PSC diagnosis up to LT or death resulting from liver failure, with censoring at last follow-up or other causes of death. The second secondary endpoint was defined as the time from PSC diagnosis up to occurrence of hepatobiliary malignancy, censored for all other preceding events, or loss to follow-up. Occurrence of hepatobiliary malignancies (*i.e.* CCA, HCC, gallbladder carcinoma [GBC], pancreatic cancer, or unclear hepatobiliary malignancy), and CRC were registered retrospectively and prospectively updated every year.

The following data on potential confounders and effect modifiers based on previous literature were collected at study inclusion and updated annually by medical chart review: age at diagnosis; sex at birth; PSC type according to the International PSC Study Group (IPSCSG) definitions[10] (*i.e.* large duct [LD], small duct [SD], or PSC with features of autoimmune hepatitis [AIH]), and concurrent diagnosis of IBD, including date and type (*i.e.* ulcerative colitis [UC], Crohn's disease [CD], or IBD unspecified [IBD-u]).[11]

Centre of inclusion was included to indicate patients originating from transplant centres and to account for potential variation within the selected patient populations. Country of residence was also collected to adjust for country-specific effects.

Statistical analysis

Given that this was an observational study, formal sample size calculation was not performed. The study encompassed all eligible participants recorded in the registry, ensuring comprehensive analysis of the available data.

Continuous data with approximately normal distribution were expressed as means ± SD, whereas skewed data were expressed as medians with IQRs. Normality of distribution was assessed visually. Categorical variables were expressed as numbers with percentages. Pearson's Chi-squared test was used to analyse categorical data. Independent *t* tests or Mann-Whitney *U* tests were used to analyse continuous data depending on whether data showed a normal or skewed distribution, respectively.

Transplant-free survival was estimated using the Kaplan–Meier method, and differences between countries were evaluated using the log-rank test. The effect of colectomy on the primary and secondary outcomes was determined via a Cox proportional hazards model using the coxph function from the survival package in R (R Foundation for Statistical Computing, Vienna, Austria).[12] Events within the first year after colectomy were censored because of a presumed delayed effect. In addition, two sensitivity analyses were performed: in the first analysis, events occurring within 1 year after colectomy were assigned to the precolectomy period. The second analysis was performed without censoring events within 1 year after colectomy, assuming the possibility of a direct effect. In the multivariable model, the effect of colectomy was corrected for the following established risk factors: sex; age at diagnosis; PSC type; and IBD status. Inclusion in a transplant centre was added as a covariate to account for variation within the selected populations. Country was added as a covariate to account for possible country-specific effects.

To account for immortal time bias, both colectomy status and IBD status were included as time-dependent exposure variables by using the counting process approach to the analysis of survival time.[13] This method stratifies the

observation time over several data records, corresponding to time intervals with constant values of all covariates.[14] The method of time-depending variables ensures that the accumulated risk in a specific risk group is adjusted for in the subsequent risk group. In case of multiple colonic surgical interventions, the time of exposure for each type of colectomy was calculated. If restorative surgery took place within 1 year after initial surgery, the type of colectomy after restoration was scored. By default, participants after proctocolectomy were coded as having no IBD. A sensitivity analysis was performed including only participants with a diagnosis of both PSC and IBD. A second sensitivity analysis was performed by including the indication for colectomy in an analysis in only participants after colectomy, retaining PSC disease duration as time axis. The rule of three was applied to estimate 95% CIs when no events were observed.[15]

Predicted survival probabilities from the final Cox proportional hazards model were used to generate predicted survival curves. The survfit function provided survival estimates with specified levels and mean values for continuous variables and were plotted by colectomy status using the ggplot2 package.[16] The results of the multivariable models per country were visualized in a forest plot using the dotwhisker package.[17] We considered p <0.05 to be statistically significant. IBM SPSS version 28.0 (IBM, Armonk, NY, USA) and R Studio 4.3.2 (RStudio, Inc. Boston, MA, USA) were used to conduct the statistical analyses.[18,19]

Results

Participant characteristics

In total, 3,110 participants were included; 1,341 from The Netherlands, 1,075 from Finland, 528 from Norway, and 166 from Sweden. Most were men (64%) with a mean age at diagnosis of 37 ± 15 years. The median calendar year of diagnosis was 2008 (IQR: 2001–2014). Of all participants, 46% were under the care of a transplant centre. Patients were diagnosed with LD PSC in 88% of cases and 75% of cases were diagnosed with IBD. Of the IBD cases, 17% were diagnosed after PSC diagnosis. The median follow-up time was 10 years, resulting in a total follow-up time of 32,236 patient-years. Participant characteristics stratified by country are presented in Table 1.

Exposure

During follow-up, 470 (15%) participants underwent colectomy; 39 (8%) had a hemicolectomy, 93 (20%) a subtotal colectomy, 295 (63%) a proctocolectomy, and 43 (9%) a colectomy of unknown extent. Among 30 participants who initially underwent a partial (*i.e.* subtotal or hemi-)colectomy, subsequent surgical intervention necessitated conversion to proctocolectomy. Of the total of 321 proctocolectomies, 232 (72%) received a pouch reconstruction and 80 (25%) received a permanent ileostomy. Active IBD was the indication for initial colectomy in 224 (48%) of participants, active disease and neoplasia in 12 (3%), low-grade dysplasia in 47 (10%), high-grade dysplasia in 45 (10%), adenocarcinoma in 85 (18%), and, in 17 (3%) of the participants, colectomy was performed because of another

Table 1. Participant characteristics stratified by country.

		IPSCR (N = 3,110)	the Netherlands (N = 1,341)	Finland (N = 1,075)	Norway (N = 528)	Sweden (N = 166)
Male, n/N (%)		1,974 /3,110 (64%)	851/1,341 (64%)	631/1,075 (59%)	382/528 (72%)	110/166 (66%)
Age at diagnosis, mean (SD)/N		37 (15)/ 3,107	39 (15)/ 1,341	36 (14)/ 1,073	39 (16)/ 528	34 (14)/ 165
Calendar year of PSC diagnosis, median (IQR)/N		2008 (2001-2014)/ 3,109	2004 (1995-2008)/ 1,341	2012 (2007-2017)/ 1,075	2011 (2007-2017)/ 528	2006 (2000-2010)/165
Follow-up time, median (IQR)/N		9 (4-15)/ 3087	9 (5-15)/ 1341	8 (4-14)/ 1073	7 (4-13)/ 508	15 (11-20)/165
Transplant centre inclusion, n/N (%)		1,437/3,110 (46%)	581/1,341 (43%)	556/1,075 (52%)	300/528 (57%)	166/166 (100%)
PSC type, n/N (%)	Large duct	2,739/3,091 (88%)	1,133/1,325 (85%)	984/1,075 (92%)	485/528 (92%)	137/163 (83%)
	Small duct	88/3,091 (3%)	79/1,325 (6%)	-	4/528 (1%)	5/163 (3%)
	Auto-immune hepatitis	264/3,091 (9%)	113/1,325 (8%)	91/1,075 (8%)	39/528 (7%)	21/163 (13%)
IBD, n/N (%)	None	774/3,110 (25%)	391/1,333 (29%)	242/1,075 (23%)	109/508 (21%)	32/164 (19%)
	Ulcerative colitis	1765/3,110 (57%)	684/1,333 (51%)	654/1,075 (61%)	325/508 (62%)	102/164 (62%)
	Crohn's disease	441/3,110 (14%)	204/1,333 (15%)	155/1,075 (14%)	55/508 (10%)	27/164 (16%)
	IBD-unspecified	100/3,110 (3%)	54/1,333 (4%)	24/1,075 (2%)	19/508 (4%)	3/164 (2%)
Colectomy*, n/N (%)	No colectomy	2639/3,110 (85%)	1154/1,341 (86%)	909/1,075 (85%)	438/528 (83%)	138/166 (83%)
	Subtotal colectomy or hemicolectomy	118/3,110 (4%)	67/1,341 (5%)	20/1,075 (2%)	27/528 (5%)	4/166 (2%)
	Proctocolectomy with pouch	230/3,110 (7%)	54/1,341 (4%)	127/1,075 (12%)	37/528 (7%)	12/166 (12%)
	Proctocolectomy with permanent ileostomy	80/3,110 (3%)	25/1,341 (2%)	18/1,075 (2%)	25/528 (5%)	12/166 (7%)
	Unknown	43/3,110 (1%)	41/1,341 (3%)	1/1,075 (0.1%)	1/528 (0.2%)	-

Participants characteristics of the IPSCR (International PSC Registry) stratified by country. SD = standard deviation. IQR = inter quartile range. PSC = primary sclerosing cholangitis. IBD = inflammatory bowel disease. Categorical data was analysed with Pearson's chi-squared test, continuous data was analysed with the independent t-test in case of normal distribution or Mann-Whitney U test in case of skewed distribution.
*Colectomy status at the end of follow-up

Table 2. Participant characteristics stratified by colectomy status.

		Participants with colectomy (N = 470)	Participants without colectomy (N = 2,640)	*p*-value
Male, n/N (%)		307/470 (65%)	1667/2,640 (63%)	0.367
Age at diagnosis, mean (SD)/N		38 (15) /470	37 (15)/ 2,637	0.117
Calendar year of PSC diagnosis, median (IQR)/N		2006 (1998-2012)/ 470	2008 (2002-2014)/ 2,639	<0.001
Follow-up time, median (IQR)/N		12 (6-19)/ 470	9 (4-14)/ 2,617	<0.001
Transplant centre inclusion, n/N (%)		230/470 (49%)	1207/2,640 (46%)	0.198
PSC type, n/N (%)	Large duct	427/469 (91%)	2312/2,622 (88%)	0.195
	Small duct	10/469 (2%)	78/2,622 (3%)	
	Auto-immune hepatitis	32/469 (7%)	232/2,622 (9%)	
IBD type, n/N (%)	None	11/469 (2%)	763/2,611 (29%)	<0.001
	Ulcerative colitis	378/469 (80%)	1387/2,611 (53%)	
	Crohn's disease	67/469 (14%)	374/2,611 (14%)	
	IBD-unspecified	13/469 (3%)	87/2,611 (3%)	

Participants characteristics for those who have undergone any type of colectomy compared to those without colectomy. SD = standard deviation. IQR = inter quartile range. PSC = primary sclerosing cholangitis. IBD = inflammatory bowel disease.
Categorical data was analysed with Pearson's chi-squared test, continuous data was analysed with the independent t-test in case of normal distribution or Mann-Whitney U test in case of skewed distribution.

indication. The indication was unknown in 33 (7%) participants.

Participant characteristics stratified by colectomy status are presented in Table 2. Participants who underwent a colectomy were more likely to have been diagnosed in significantly earlier years compared with those without colectomy (median 2006 *vs.* 2008, p <0.001). Consequently, follow-up duration differed significantly between participants with and without colectomy, with median follow-up periods of 12 years and 9 years (p <0.001), respectively. In addition, the distribution of IBD type differed significantly between participants with and without colectomy, with fewer participants without IBD (2% *vs.* 29%, p <0.001) and a higher proportion of UC (80% *vs.* 53%, p <0.001) in the group who underwent colectomy.

Primary outcome

In total, we observed 395 (13%) deaths, of which 290 (73%) were PSC related. Cause of death was missing for 38 (10%) participants. Of the PSC-related deaths, 77 (27%) participants died from liver failure, 176 (61%) from hepatobiliary cancer, 20 (7%) from CRC, and 11 (4%) from other PSC-related causes. Of the 653 (21%) LTs observed, 237 (36%) were performed because of end-stage liver disease, 46 (7%) because of recurrent bacterial cholangitis, 28 (4%) because of itching or fatigue, 11 (2%) because of CCA, 7 (1%) because of HCC, and 48 (7%) because of biliary dysplasia. Indication for LT was missing for 274 (42%) participants.

The median transplant-free survival time was 22 (95% CI: 21–23) years. Transplant-free survival differed significantly between countries, with a median of 19 (95% CI: 17–20) years for The Netherlands, 23 (95% CI: 19–31) years for Norway, 29 (95% CI: 25–N/A) years for Finland, and 33 (95% CI: 24–N/A) years for Sweden. Survival curves stratified per country are presented in Fig. S1. Median time from colectomy to last follow-up date or endpoint was 5 (1–11) years.

Transplant-free survival was analysed based on colectomy status and specified by extent (*i.e.* hemicolectomy, subtotal colectomy, proctocolectomy with pouch, or proctocolectomy with permanent ileostomy). The results of the univariable and multivariable models are presented in Table 3. There was a clear separation in hazard rate of endpoints regarding extent of colectomy in univariable analyses, with lowest risk for proctocolectomy with ileostomy. For the endpoint LT or PSC-related death (excluding CRC), proctocolectomy with permanent ileostomy remained the most notable predictor (HR 0.41; 95%:CI: 0.24–0.71). Subtotal colectomy or hemicolectomy and proctocolectomy with pouch were not significantly associated with survival (HR 0.81; 95% CI: 0.58–1.12; and 1.00; 95% CI:0.73–1.38), respectively). Survival differed significantly between proctocolectomy with ileostomy and proctocolectomy with pouch (p = 0.003) and between proctocolectomy with ileostomy and subtotal colectomy or hemicolectomy (p = 0.04). In the multivariable model for the endpoint LT or all-cause mortality, adjusted for sex, age, PSC type, IBD type, country, and inclusion in a transplant centre, patients with proctocolectomy with ileostomy had a significantly decreased hazard compared with patients with intact colon (HR 0.48; 95% CI: 0.28–0.80)). Subtotal colectomy or hemicolectomy or proctocolectomy with pouch were not associated with a significantly decreased hazard (HR 1.13; 95% CI: 0.85–1.50; and 0.80; 95% CI: 0.59–1.08), respectively).

In line with previous literature, male sex, older age at diagnosis, and inclusion in a transplant centre were associated with reduced survival outcomes. Among the IBD diagnoses, these were associated in the multivariable analysis with reduced HRs for the endpoint LT or all-cause mortality but not for LT or PSC-related death. The predicted survival curve for a 37-year-old male patient with LD PSC and UC in The Netherlands, modelled as though the participant had undergone a specific type of colectomy at the beginning and maintained that status throughout the follow-up period, is presented in Fig. 1. The predicted median survival time for those with proctocolectomy with ileostomy was 35 years compared with 19 years for those without colectomy.

The model was also applied to each country individually. The results of the individual models are presented in Table S1 and depicted in a forest plot in Fig. 2. Predicted survival curves are presented per country in Fig. S2. Despite some variation between countries, a consistent beneficial effect was estimated in the association between proctocolectomy with permanent ileostomy and transplant-free survival in three out of four countries. The HRs were 0.43 (95% CI: 0.21–0.90) in The Netherlands, 0.98 (95% CI: 0.13–7.27) in Finland, 0.33 (95%

Table 3. Multivariable model results for transplant-free survival, defined as liver transplantation or PSC-related death (excluding CRC) (A); and liver transplantation or all-cause mortality (B).

A. Endpoint liver transplantation or PSC-related death (excluding CRC)		**Univariable HR (95% CI)**	**Multivariable HR (95% CI)**
Colectomy	*None*	Reference	Reference
	Subtotal colectomy or hemicolectomy	1.18 (0.85-1.63)	0.81 (0.58-1.12)
	Proctocolectomy with pouch	1.02 (0.76-1.36)	1.00 (0.73-1.38)
	Proctocolectomy with ileostomy	0.62 (0.37-1.06)	0.41 (0.24-0.71)
Female		0.82 (0.72-0.95)	0.79 (0.68-0.91)
Age at diagnosis (per year)		1.02 (1.02-1.03)	1.02 (1.02-1.03)
PSC type	*Large duct*	Reference	Reference
	Small duct	0.26 (0.13-0.53)	0.22 (0.11-0.45)
	Features of AIH	1.03 (0.82-1.29)	1.07 (0.85-1.35)
IBD	*No IBD*	Reference	Reference
	Ulcerative colitis	0.98 (0.85-1.14)	1.01 (0.86-1.20)
	Crohn's disease	0.77 (0.62-0.97)	0.80 (0.63-1.01)
	IBD-unspecified	0.91 (0.61-1.36)	0.96 (0.64-1.45)
Country	*the Netherlands*	Reference	Reference
	Finland	0.43 (0.31-0.58)	0.59 (0.43-0.81)
	Norway	0.87 (0.74-1.04)	0.78 (0.65-0.93)
	Sweden	0.41 (0.34-0.49)	0.36 (0.30-0.44)
Transplant centre patient		1.65 (1.44-1.88)	1.80 (1.57-2.08)
B. Endpoint liver transplantation or all-cause mortality		**Univariable HR (95% CI)**	**Multivariable HR (95% CI)**
Colectomy	*None*	Reference	Reference
	Subtotal colectomy or hemicolectomy	1.73 (1.31-2.29)	1.13 (0.85-1.50)
	Proctocolectomy with pouch	0.97 (0.74-1.28)	0.80 (0.59-1.08)
	Proctocolectomy with ileostomy	0.68 (0.41-1.12)	0.48 (0.28-0.80)
Female		0.89 (0.78-1.02)	0.83 (0.72-0.95)
Age at diagnosis (per year)		1.03 (1.03-1.04)	1.03 (1.02-1.03)
PSC type	*Large duct*	Reference	Reference
	Small duct	1.48 (0.89-2.46)	1.06 (0.63-1.77)
	Features of AIH	1.01 (0.81-1.26)	1.15 (0.91-1.44)
IBD	*No IBD*	Reference	Reference
	Ulcerative colitis	0.80 (0.69-0.92)	0.75 (0.64-0.88)
	Crohn's disease	0.67 (0.54-0.83)	0.69 (0.55-0.86)
	IBD-unspecified	0.79 (0.53-1.18)	0.62 (0.41-0.93)
Country	*the Netherlands*	Reference	Reference
	Finland	0.21 (0.16-0.28)	0.26 (0.19-0.35)
	Norway	0.41 (0.35-0.49)	0.42 (0.35-0.50)
	Sweden	0.20 (0.17-0.24)	0.20 (0.17-0.25)
Transplant centre patient		1.23 (1.08-1.39)	1.33 (1.16-1.52)

Univariable and multivariable outcomes of the Cox proportional hazards model for the outcomes (A) liver transplantation or all-cause mortality and (B) liver transplantation or PSC-related death (excluding CRC). The table summarized the hazard ratios (HR) and 95% confidence intervals (95%CI) for colectomy status, sex, age at diagnosis, PSC type, IBD status, country and centre of inclusion.

CI: 0.13–0.86) in Norway, and 0.00 (95% CI: 0.00–0.25) in Sweden, the latter resulting from no events under 137 person-years in the proctocolectomy with permanent ileostomy group.

Sensitivity analysis

First, we performed two sensitivity analyses to test the effect of the presumed delayed effect of colectomy of 1 year. We started by modelling a delay of 1 year, which implicates that events within the first year after colectomy were assigned to the pre-colectomy period (Table S2). The adjusted HRs were 0.91 (95% CI: 0.67–1.25) for subtotal colectomy or hemicolectomy, 1.09 (95% CI: 0.81–1.48) for proctocolectomy with pouch, and 0.47 (95% CI: 0.28–0.79) for proctocolectomy with ileostomy. We also computed a model without censoring of events within 1 year after colectomy. The adjusted HRs were 0.94 (95% CI: 0.69–1.27) for subtotal colectomy or hemicolectomy, 1.09 (95% CI: 0.81–1.49) for proctocolectomy with pouch, and 0.50 (95% CI: 0.30–0.83) for proctocolectomy with ileostomy.

Second, we performed a sensitivity analysis including only participants with a diagnosis of both PSC and IBD (Table S3). Compared with the analysis including all participants, the effect of colectomy was more pronounced for all subtypes. The adjusted HRs were 0.76 (95% CI: 0.53–1.09) for subtotal colectomy or hemicolectomy, 0.55 (95% CI: 0.24–1.27) for proctocolectomy with pouch, and 0.24 (95% CI: 0.09–0.61) for proctocolectomy with ileostomy.

Third, we performed a sensitivity analysis on two secondary endpoints to distinguish between outcomes driven by the progression of liver disease *vs.* hepatobiliary cancer. In the first analysis, which focused on the liver-specific outcome (Table S4A), the adjusted HRs were 0.65 (95% CI: 0.43–0.97) for subtotal colectomy or hemicolectomy, 0.75 (95% CI: 0.50–1.11) for proctocolectomy with pouch, and 0.24 (95% CI: 0.10–0.53) for proctocolectomy with ileostomy. The second analysis focused on occurrence of hepatobiliary malignancies (Table S4B). In total, we observed 202 (7%) cases of CCA, 34 (1%) of GBC, 38 (1%) of HCC, 5 (0.2%) of pancreatic cancer, and 1 (0.05%) case of hepatobiliary malignancy of unclear origin. The adjusted HRs were 1.26 (95% CI: 0.75–2.13) for subtotal colectomy or hemicolectomy, 1.62 (95% CI: 0.96–2.75) for proctocolectomy with pouch, and 1.17 (95% CI: 0.60–2.29) for proctocolectomy with ileostomy.

Lastly, a sensitivity analysis in participants after colectomy was performed to assess the effect of surgical indication. No

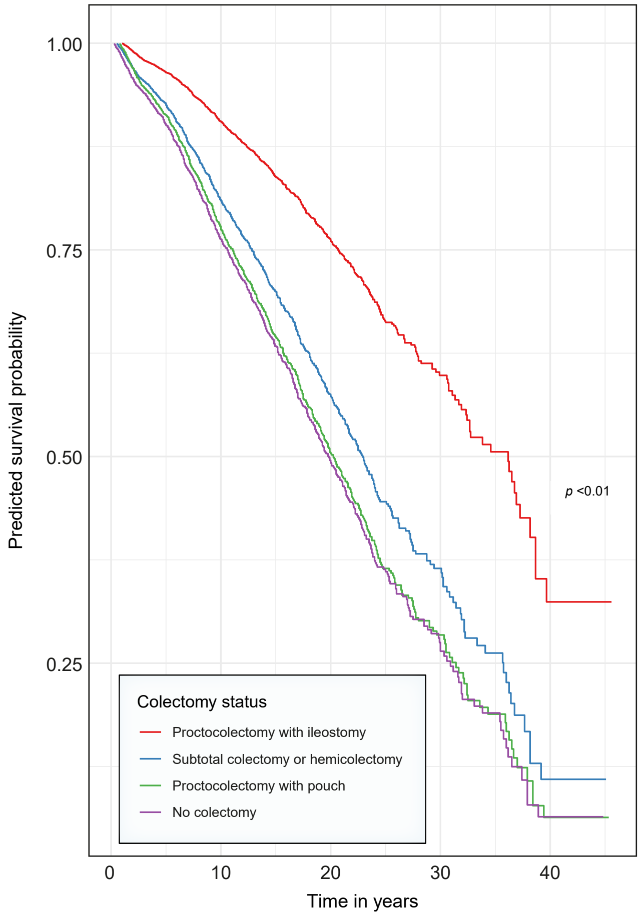

Fig. 1. Predicted survival probability for a 37-year-old man with LD PSC and UC from The Netherlands for the endpoint LT or PSC-related death (excluding CRC). The Kaplan–Meier curves demonstrate the estimated survival probabilities over time for participants without colectomy (red line), participants after subtotal colectomy or hemicolectomy (green line), participants after proctocolectomy with pouch (blue line), and participants after proctocolectomy with ileostomy (purple line). Survival probabilities (y-axis) are modelled under the assumption that a participant maintains their colectomy status throughout their follow-up period (x-axis; years since PSC diagnosis). CRC colorectal cancer; LD, large duct; LT, liver transplantation; PSC, primary sclerosing cholangitis; UC, ulcerative colitis.

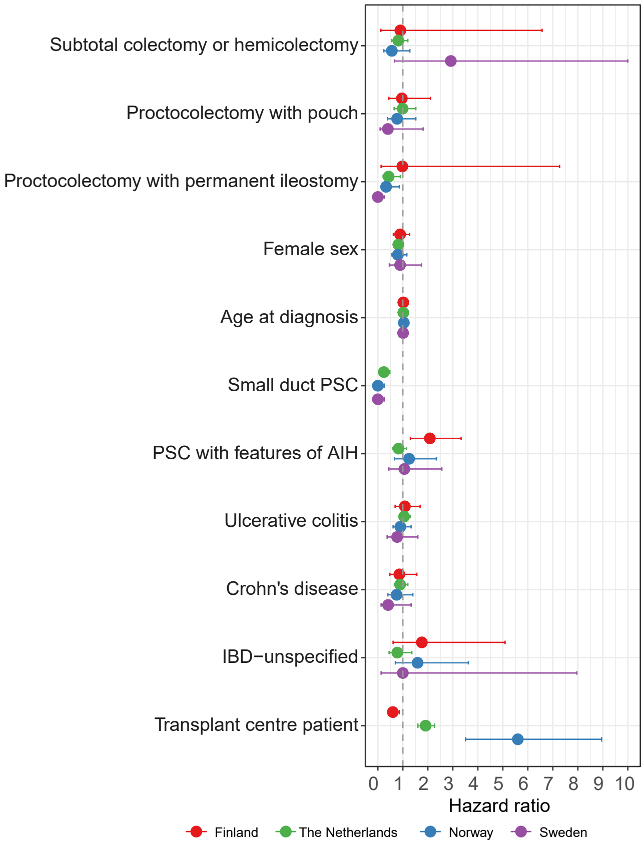

Fig. 2. Results of the multivariable models stratified per country. The forest plot illustrates the outcomes of the multivariable model for the endpoint LT or PSC-related death (excluding CRC) per country. The variables included in the model are listed on the y-axis, the dots on the x-axis represent the HR for every variable from the country-specific model, including whiskers presenting the 95% CIs. The outcomes for the Finnish model are in red, the outcomes for the model from The Netherlands are in green, the outcomes for the Norwegian model are in blue, and the outcomes for the Swedish model are in purple. The dotted line depicts an HR of 1. AIH, autoimmune hepatitis; CRC colorectal cancer; HR, hazard ratio; IBD, inflammatory bowel disease; LT, liver transplantation; PSC, primary sclerosing cholangitis.

significant effect of surgical indication was observed (Table S5). However, the HR for participants after proctocolectomy with pouch was significantly increased compared with those after proctocolectomy with ileostomy (HR 2.01; 95% CI: 1.04–3.88).

Discussion

Our study assesses the effect of colectomy on the disease course of PSC in an international cohort, accounting for time dependency and established risk factors. Our findings indicate that proctocolectomy with permanent ileostomy is associated with a decreased risk of LT and PSC-related death. This effect was mainly associated with postponement of LT and death from liver failure, as shown by the analyses of the secondary outcomes. In the subset of participants with a diagnosis of both PSC and IBD at any time during their follow-up, the effect of colectomy was even more pronounced for all subtypes. Surgical indication did not appear to affect transplant-free survival in those after colectomy. Three additional sensitivity-analysis outcomes also added robustness to the findings.

To date, most studies have concentrated on the effect of colectomy on outcomes following LT, whereas limited evidence exists regarding its effect on disease progression in the pretransplant phase. Ong *et al.*[8] evaluated all available evidence on PSC progression and transplant-free survival in patients who underwent colectomy. In their systematic review, no clear effect on solid clinical endpoints, such as LT or mortality, was found. The conclusions of the largest study included in this systematic review, comprising almost 20,000 patient-years follow-up, are partly in line with our findings. The authors reported a significant association between participants treated with colectomy before PSC diagnosis and a decreased risk of LT and death, however, this effect was not found when colectomy was performed after PSC diagnosis.[20] Given that the study by Nordenvall *et al.*[20] was based on Swedish healthcare registers, case verification to ensure minimal case omission and misdiagnosis could not be performed. Moreover, detailed clinical information was not available to correct for PSC type, or stratify for different types of colectomy.

Furthermore, the other included studies were based on small sample sizes and their statistical analyses did not correct for a time-depending effect of colectomy.

A few studies have been published describing the effect of colectomy on post-transplant outcomes. A meta-analysis of the report by Steenstraten *et al.*[6] demonstrated a significant beneficial effect of colectomy before LT on the risk of recurrent PSC (HR 0.65; 95% CI: 0.42–0.99). In addition, studies from Trivedi *et al.*[7] and Matar *et al.*[21] included details regarding the extent and type of colectomy and demonstrated a beneficial effect of proctocolectomy with permanent ileostomy compared with proctocolectomy with pouch reconstruction on PSC recurrence rates. The study by Schabl *et al.*[22] could not confirm the beneficial effect of ileostomy, although this analysis was likely underpowered, given that it included only nine participants with an ileostomy.

The strong association between proctocolectomy with permanent ileostomy and transplant-free survival could be explained by various hypotheses. Inflammation in the remaining colon or pouch could lead to persistent translocation of pathogens according to the leaky gut hypothesis and ongoing gut lymphocyte homing in the liver. Unfortunately, data regarding pouchitis were not available in our database. PSC is a strong risk factor for pouchitis.[23] Following pouch reconstruction, the mucosa and microbiome of the pouch change significantly and adapt to a more colon-like phenotype.[24,25] This transformation does not occur in ileal mucosa after ileostomy and appears inherent to stasis in the pouch. Morphological alterations are most predominant in those with pouchitis compared with normal pouches and might be dependent on the microbiota composition.[23,26] Moreover, pouchitis does not occur before exposure to faecal contents, underscoring the putative role of the microbiota.

Our findings contribute to our understanding of the aetiology of PSC. The gut–liver axis appears to have a role in the pathophysiology of PSC and deserves focus as therapeutic target for PSC management. Therapeutic strategies, such as faecal microbiota transplantation (FMT), could hold promise in modulating gut microbiota and, as a result, could modify PSC progression. Given the potential complications associated with pouch reconstruction in patients with PSC and the beneficial long-term outcomes associated with ileostomy, it might be prudent to consider proctocolectomy with permanent ileostomy when colectomy is indicated. Quality of life (QoL) studies in patients with ileostomy are limited, but available evidence suggests that QoL outcomes are not substantially different between those with pouch or ileostomy.[27] This consideration should be discussed with patients with PSC when making surgical decisions.

Our study presents the largest cohort to date describing the effect of colectomy on disease progression of PSC, a situation that cannot be studied in a randomised controlled fashion. It demonstrates the merits of mining data from our large uniform EASL-funded IPSCR, enabling detailed clinical data to be included for the first time to correct for established risk factors and stratification for colectomy extent, as well as correction for the time-dependent effect of colectomy.

Several limitations must be addressed. Given our observational study design, we cannot rule out unknown or overlooked confounders that might influence our outcomes. The effect of colectomy was similar among cohorts in three out of four countries. Proctocolectomy with permanent ileostomy showed no apparent benefit in Finland, likely because of local guidelines reserving ileostomy for pouch failure or technical impossibility of pouch reconstruction. It might be that, because of a high inflammatory burden after pouch failure, the effect of ileostomy was limited in this patient population. Moreover, we observed heterogeneity across countries regarding overall survival and effect of covariates, especially for the effect of participants under care in a transplantation centre. This is most likely attributable to patient selection and national practice guidelines. Although the amount of missing data overall was small, the cause of death and indication for LT were missing in a substantial percentage of participants. These were mostly participants in the Dutch population-based cohort, for whom data could not be retrieved because of GDPR regulations. Moreover, data completeness might have been impacted by the retrospective nature of data collection. The estimated effect of colectomy was similar for the endpoint LT or all-cause mortality and LT and PSC-related death (excluding CRC). Therefore, the risk of informative censoring in this selection of participants is expected to be low. Prognostic markers of liver disease severity were not available for all participants at time of PSC diagnosis and, therefore, were not included in the analyses.

In conclusion, our data indicate a beneficial effect of proctocolectomy with permanent ileostomy on liver disease progression in patients with PSC-IBD. These findings contribute to our understanding of the pathogenesis of PSC, could fuel novel therapeutic strategies, and call for reconsideration in counselling patients awaiting proctocolectomy.

Affiliations

[1]*Department of Gastroenterology and Hepatology, Amsterdam University Medical Centre, Amsterdam, The Netherlands;* [2]*Amsterdam Gastroenterology Endocrinology Metabolism, Amsterdam, The Netherlands;* [3]*Department of Gastroenterology and Hepatology, Helsinki University Hospital, Helsinki, Finland;* [4]*Norwegian PSC Research Centre, Department of Transplantation Medicine, Oslo University Hospital, Oslo, Norway;* [5]*Institute of Clinical Medicine, University of Oslo, Oslo, Norway;* [6]*Research Institute of Internal Medicine, Division of Surgery and Specialized Medicine, Oslo University Hospital, Oslo, Norway;* [7]*Section of Gastroenterology, Department of Transplantation Medicine, Oslo University Hospital, Oslo, Norway;* [8]*Department of Medicine, Haraldsplass Deaconess Hospital and Department of Clinical Science, University of Bergen, Bergen, Norway;* [9]*Department of Gastroenterology, Akershus University Hospital, Lørenskog, Norway;* [10]*Unit of Gastroenterology and Rheumatology, Department of Medicine Huddinge, Karolinska Institutet, Karolinska University Hospital, Stockholm, Sweden;* [11]*Department of Gastroenterology and Hepatology, Erasmus Medical Centre, Rotterdam, The Netherlands;* [12]*Department of Gastroenterology and Hepatology, University Medical Centre Groningen, Groningen, The Netherlands;* [13]*Department of Gastroenterology and Hepatology, Leiden University Medical Centre, Leiden, The Netherlands;* [14]*Department of Epidemiology and Data Science, Amsterdam University Medical Centre, Amsterdam, The Netherlands;* [15]*Amsterdam Institute for Immunology and Infectious diseases (AI&I), Amsterdam, The Netherlands*

Abbreviations

AIH, autoimmune hepatitis; CCA, cholangiocarcinoma; CD, Crohn's disease; CRC, colorectal carcinoma; FMT, faecal microbiota transplantation; GBC, gallbladder cancer; HCC, Hepatocellular carcinoma; HR, hazard ratio; IBD, inflammatory bowel disease; IBD-U, inflammatory bowel disease – unspecified; IPSCSG, International PSC Study Group; IPSCR, International PSC Registry; LD, large duct; LT, liver transplantation; PSC, primary sclerosing cholangitis; QoL, quality of life; SD, small duct; UC, ulcerative colitis.

Financial support

The study was funded by a registry grant from EASL, the Dutch Organisation for Health Research and Development (ZonMw, grant number:836041010), and a grant from Gilead. The funders of the study had no role in study design, data collection, data analysis, data interpretation, or writing of the report.

Conflicts of interest

The authors have no financial or personal relationships to declare that are relevant to this publication.

Please refer to the accompanying ICMJE disclosure forms for further details.

Authors' contributions

Conceived the idea for this study: BM, KM, JB, CP. Data collection: BM, MN, KM, MF, TF, ST, JH, KB, MV, KJ, AB, AV, AM, RW, AI, CP. Verification of underlying data: BM, MN. Statistical analyses: BM, MN, JB. All authors were involved in critical revision of the manuscript for important intellectual content. All authors had full access to all the data and accepted responsibility to submit for publication.

Data availability

Data are available upon reasonable request from the corresponding author. Deidentified data are available from the corresponding author after approval of a proposal by the IPSCR steering committee and with signed data access agreement.

Supplementary data

Supplementary data to this article can be found online at https://doi.org/10.1016/j.jhepr.2025.101700.

References

[1] van Munster KN, Bergquist A, Ponsioen CY. Inflammatory bowel disease and primary sclerosing cholangitis: one disease or two? J Hepatol 2024;80:155–168.
[2] Berg RD. Bacterial translocation from the gastrointestinal tract. In: Paul PS, Francis DH, editors. Mechanisms in the pathogenesis of enteric diseases 2. Boston: Springer US; 1999. p. 11–30.
[3] Grant AJ, Lalor PF, Salmi M, et al. Homing of mucosal lymphocytes to the liver in the pathogenesis of hepatic complications of inflammatory bowel disease. Lancet 2002;359:150–157.
[4] Hov JR, Karlsen TH. The microbiota and the gut-liver axis in primary sclerosing cholangitis. Nat Rev Gastroenterol Hepatol 2023;20:135–154.
[5] Zheng HH, Jiang XL. Increased risk of colorectal neoplasia in patients with primary sclerosing cholangitis and inflammatory bowel disease: a meta-analysis of 16 observational studies. Eur J Gastroenterol Hepatol 2016;28:383–390.
[6] Steenstraten IC, Sebib Korkmaz K, Trivedi PJ, et al. Systematic review with meta-analysis: risk factors for recurrent primary sclerosing cholangitis after liver transplantation. Aliment Pharmacol Ther 2019;49:636–643.
[7] Trivedi PJ, Reece J, Laing RW, et al. The impact of ileal pouch-anal anastomosis on graft survival following liver transplantation for primary sclerosing cholangitis. Aliment Pharmacol Ther 2018;48:322–332.
[8] Ong J, Bath MF, Swift C, et al. Does colectomy affect the progression of primary sclerosing cholangitis? A systematic review and meta-analysis. Gastroenterol Hepatol Bed Bench 2018;11:277–283.
[9] European Association for the Study of the Liver. EASL clinical practice guidelines on sclerosing cholangitis. J Hepatol 2022;77:761–806.
[10] Ponsioen CY, Assis DN, Boberg KM, et al. Defining primary sclerosing cholangitis: results from an international primary sclerosing cholangitis study group consensus process. Gastroenterology 2021;161:1764–1775.
[11] Weismüller TJ, Trivedi PJ, Bergquist A, et al. Patient age, sex, and inflammatory bowel disease phenotype associate with course of primary sclerosing cholangitis. Gastroenterology 2017;152:1975–1984.
[12] Terry M, Therneau PMG. Modeling survival data: extending the Cox model. R package version35–7. New York: Springer; 2000.
[13] Hosmer DW, Lemeshow S, May S. A*pplied survival analysis. Regression Modeling of time-to-event data*. 2nd edn. Hoboken: John Wiley & Sons; 2008.
[14] Andersen PK, Gill RD. Cox's regression model for counting processes: a large sample study. Ann Stat 1982;10:1100–1120.
[15] Hanley JA, Lippman-Hand A. If nothing goes wrong, is everything all right? Interpreting zero numerators. JAMA 1983;249:1743–1745.
[16] Wickham H. ggplot2: elegant graphics for data analysis. New York: Springer-Verlag; 2016.
[17] Solt F. Hu Y. Dotwhisker: dot-and-whisker plots of regression results. https://cran.r-project.org/web/packages/dotwhisker/vignettes/dotwhisker-vignette.html [Accessed 01 December 2025].
[18] Team R. RStudio. Integrated development for R. Boston, MA: RStudio; 2020.
[19] IBM Corp. IBM SPSS statistics for windows, version 28.0. Armonk, NY: IBM Corp; 2021.
[20] Nordenvall C, Olén O, Nilsson PJ, et al. Colectomy prior to diagnosis of primary sclerosing cholangitis is associated with improved prognosis in a nationwide cohort study of 2594 PSC-IBD patients. Aliment Pharmacol Ther 2018;47:238–245.
[21] Matar AJ, Falconer E, LaBella M, et al. Long-term outcomes following colectomy and liver transplantation for inflammatory bowel disease with primary sclerosing cholangitis. Ann Surg 2024. https://doi.org/10.1097/SLA.0000000000006533. Published online September 11.
[22] Schabl L, Holubar SD, Maspero M, et al. Ileal pouch-anal anastomosis and end ileostomy result in equivalent graft survival following liver transplantation for inflammatory bowel disease-primary sclerosing cholangitis. Tech Coloproctol 2024;28:113.
[23] Barnes EL, Holubar SD, Herfarth HH. Systematic review and meta-analysis of outcomes after ileal pouch-anal anastomosis in primary sclerosing cholangitis and ulcerative colitis. J Crohns Colitis 2021;15:1272–1278.
[24] de Silva HJ, Millard PR, Kettlewell M, et al. Mucosal characteristics of pelvic ileal pouches. Gut 1991;32:61–65.
[25] Young VB, Raffals LH, Huse SM, et al. Multiphasic analysis of the temporal development of the distal gut microbiota in patients following ileal pouch anal anastomosis. Microbiome 2013;1:9.
[26] Machiels K, Sabino J, Vandermosten L, et al. Specific members of the predominant gut microbiota predict pouchitis following colectomy and IPAA in UC. Gut 2017;66:79–88.
[27] Kuruvilla K, Osler T, Hyman NH. A comparison of the quality of life of ulcerative colitis patients after IPAA vs ileostomy. Dis Colon Rectum 2012;55:1131–1137.

Keywords: Primary sclerosing cholangitis; Inflammatory bowel disease; Colectomy.
Received 10 May 2025; received in revised form 17 November 2025; accepted 24 November 2025; Available online 22 December 2025

Journal of Hepatology, Volume *8*

Supplemental information

Proctocolectomy with permanent ileostomy is associated with improved transplant-free survival in patients with PSC

Bregje Mol, Moyrha van Nieuwamerongen, Kim N. van Munster, Martti Färkkilä, Trine Folseraas, Sara K.V. Tjønnfjord, Johannes R. Hov, Kirsten Boberg, Mette Vesterhus, Kristin K. Jørgensen, Annika Bergquist, Jorn C. Goet, Annemarie C. de Vries, Adriaan J.P. van der Meer, Rinse K. Weersma, Akin Inderson, Johannes A. Bogaards, Cyriel Y. Ponsioen, and on behalf of the IPSCR study group

Proctocolectomy with permanent ileostomy is associated with improved transplant-free survival in patients with PSC

Bregje Mol, Moyrha van Nieuwamerongen, Kim van Munster, Martti Färkkilä, Trine Folseraas, Sara Tjønnfjord, Johannes Hov, Kirsten Boberg, Mette Vesterhus, Kristin Jørgensen, Annika Bergquist, Jorn Goet, Annemarie de Vries, Adriaan van der Meer, Rinse Weersma, Akin Inderson, Johannes Bogaards, Cyriel Ponsioen on behalf of the IPSCR study group

Table of contents

Supplementary file……………………………………………………………………..2

Fig. S1…………………………………………………………………………………….3

Table S1………………………………………………………………………….......8

Fig. S2……………………………………………………………………………………12

Table S2………………………………………………………………………….....13

Table S3………………………………………………………………………….....15

Table S4………………………………………………………………………….....17

Table S5………………………………………………………………………….....19

Table S6………………………………………………………………………….....20

Supplementary references…………………………………………………..……..….21

Supplementary File 1. Description of the separate cohorts

The Finnish cohort consists of patients retrieved from the Helsinki University Hospital (HUH) PSC-registry, Finland. HUH is a referral centre responsible for the Helsinki and Uusimaa hospital district, comprising 39% of the Finnish population. All patients referred to HUH for a diagnostic, follow-up, or surveillance ERCP were recruited from 2009 onward. Data were collected up to liver transplantation, death or January 2024.

The Dutch cohort consists of patients derived from 46 hospitals participating in the national dynamic EpiPSC2 registry. The registry was initiated in 2008 as the population based EpiPSC/PBC study and transitioned to the prospective EpiPSC2 study in 2016. All eligible participants, in compliance with GDPR, were invited to participate in the prospective arm of the study. Patients were followed up to liver transplantation, death or data lock in January 2022. Clinical data were sourced from local electronic hospital databases and written correspondence from treating physicians. All patients participating in the prospective arm of the study provided written informed consent.

The Norwegian cohort consists of patients with PSC prospectively recruited at admission to the tertiary care hospital Oslo University Hospital, Rikshospitalet (Oslo, Norway) between 2008 and 2022. Additionally, patients were selected from the prospective NoPSC national database. All non-transplanted patients with a diagnosis of PSC at baseline were included in the prospective NoPSC national database by May 2023 from Oslo University Hospital Rikshospitalet, Oslo; Akershus University Hospital, Oslo; and Harladsplass Deaconess Hospital, Bergen. Clinical follow-up data was collected up to liver transplantation, death or May 2023.

The Swedish cohort consists of patients from the Karolinska University hospital Huddinge included in the Swedish prospective surveillance study. Patients were included between November 2011 and April 2016 in a 5-year surveillance programme. Exclusion criteria were expected listing for liver transplantation within one year, previously transplanted patients and presence of a hepatobiliary malignancy. Patients were followed up to liver transplantation, death or August 2023. Clinical data were obtained by the treating physician. All patients provided written informed consent.

Fig. S1. Survival plots of all patients stratified per country for the endpoint LT or PSC-related death (excluding CRC).

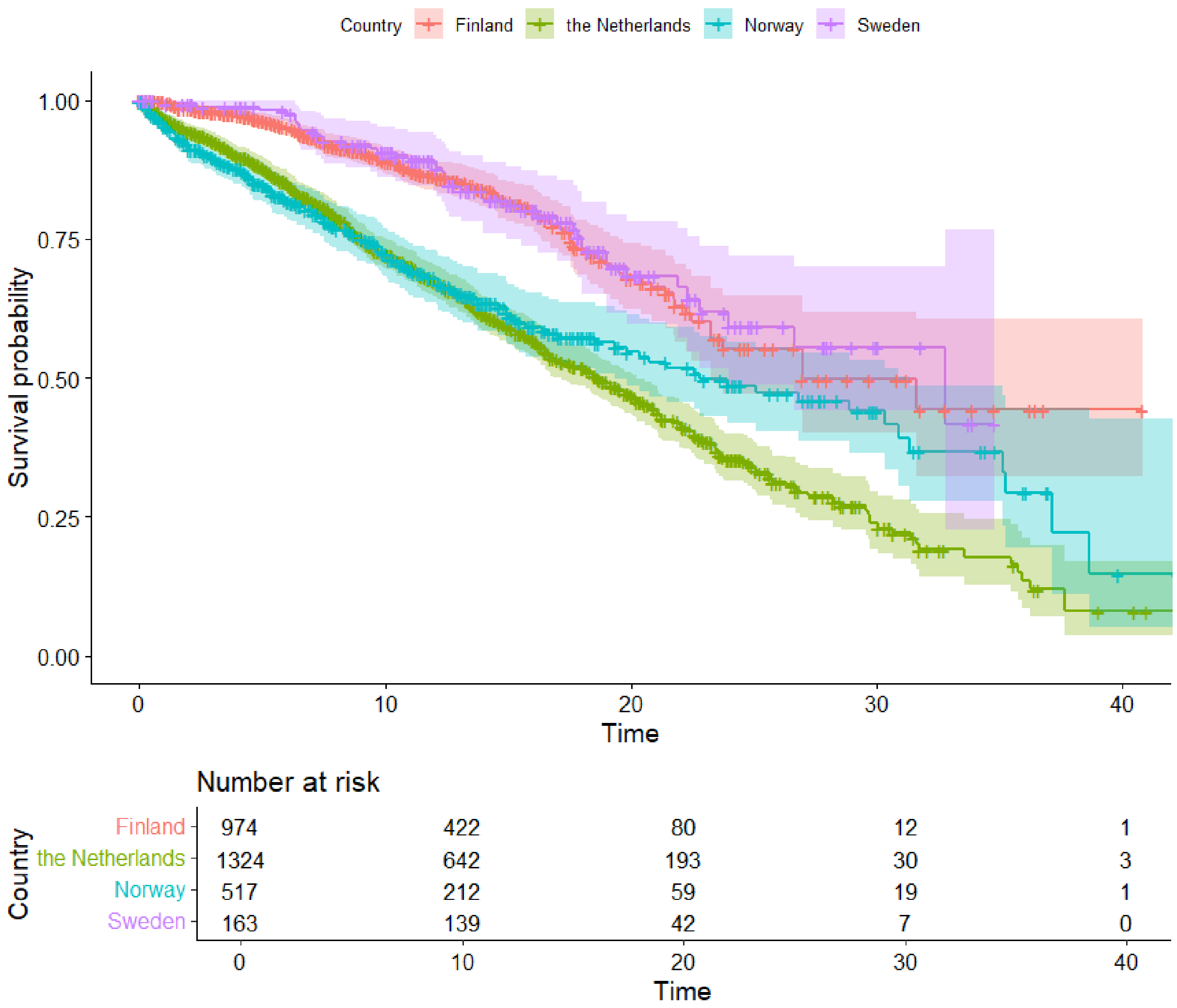

Table S1. Multivariable model results for transplant-free survival defined as time up to liver transplantation of PSC-related death (excluding colorectal carcinoma) stratified per country

A. Finland		HR (95%CI)
Colectomy	*None*	Reference
	Subtotal colectomy or hemicolectomy	0.90 (0.12-6.57)
	Proctocolectomy with pouch	0.96 (0.44-2.11)
	Proctocolectomy with ileostomy	0.98 (0.13-7.27)
Female		0.89 (0.62-1.27)
Age at diagnosis (per year)		1.02 (1.01-1.04)
PSC type	*Large duct*	Reference
	Small duct	N.A.
	Features of AIH	2.08 (1.30-3.33)
IBD	*No IBD*	Reference
	Ulcerative colitis	1.08 (0.69-1.69)
	Crohn's disease	0.86 (0.48-1.56)
	IBD-unspecified	1.76 (0.61-5.09)
Transplant centre patient		0.60 (0.42-0.86)
B. The Netherlands		HR (95%CI)
Colectomy	*None*	Reference
	Subtotal colectomy or hemicolectomy	0.82 (0.56-1.20)

	Proctocolectomy with pouch<br>*Proctocolectomy with ileostomy*	0.99 (0.65-1.52)<br>0.43 (0.21-0.90)
Female		0.82 (0.68-0.99)
Age at diagnosis (per year)		1.02 (1.01-1.02)
PSC type	*Large duct*<br>*Small duct*<br>*Features of AIH*	Reference<br>0.23 (0.12-0.48)<br>0.83 (0.60-1.15)
IBD	*No IBD*<br>*Ulcerative colitis*<br>*Crohn's disease*<br>*IBD-unspecified*	Reference<br>1.04 (0.84-1.28)<br>0.88 (0.65-1.18)<br>0.76 (0.44-1.33)
Transplant centre patient		1.90 (1.60-2.26)
C. Norway		HR (95%CI)
Colectomy	*None*<br>Subtotal colectomy or hemicolectomy<br>*Proctocolectomy with pouch*<br>*Proctocolectomy with ileostomy*	Reference<br>0.56 (0.24-1.28)<br>0.77 (0.39-1.52)<br>0.33 (0.13-0.86)
Female		0.80 (0.56-1.16)
Age at diagnosis (per year)		1.04 (1.03-1.05)

PSC type	*Large duct*<br>*Small duct*<br>*Features of AIH*	Reference<br>0.00 (0.00-N.A.)<br>1.25 (0.67-2.35)
IBD	*No IBD*<br>*Ulcerative colitis*<br>*Crohn's disease*<br>*IBD-unspecified*	Reference<br>0.90 (0.61-1.33)<br>0.75 (0.40-1.40)<br>1.59 (0.70-3.63)
Transplant centre patient		5.61 (3.51-8.96)
D. Sweden		HR (95%CI)
Colectomy	*None*<br>Subtotal colectomy or hemicolectomy<br>*Proctocolectomy with pouch*<br>*Proctocolectomy with ileostomy*	Reference<br>2.92 (0.67-12.66)<br>0.40 (0.09-1.81)<br>0.00 (0.00-N.A.)
Female		0.89 (0.46-1.75)
Age at diagnosis (per year)		1.01 (0.99-1.04)
PSC type	*Large duct*<br>*Small duct*<br>*Features of AIH*	Reference<br>0.00 (0.00-N.A.)<br>1.06 (0.44-2.56)
IBD	*No IBD*<br>*Ulcerative colitis*	Reference<br>0.77 (0.37-1.60)

	Crohn's disease<br>*IBD-unspecified*	0.41 (0.13-1.33)<br>1.00 (0.13-7.96)
Transplant centre patient		N.A.

Fig. S2A-D. Predicted survival probabilities for a 37 year-old male with large duct PSC and ulcerative colitis from the Netherlands (A), Finland (B), Norway (C), Sweden (D) for the endpoint LT or PSC-related death (excluding CRC).

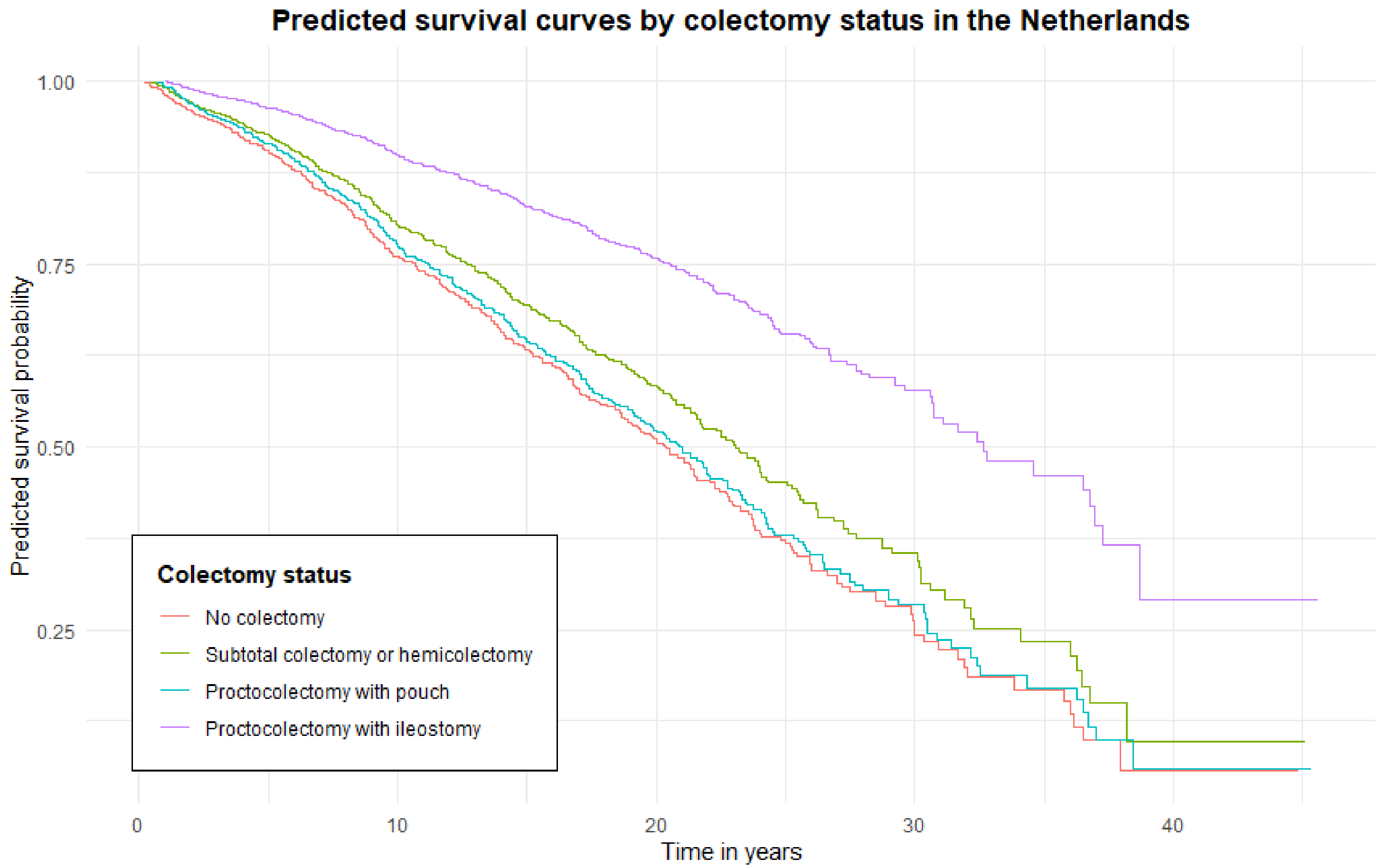

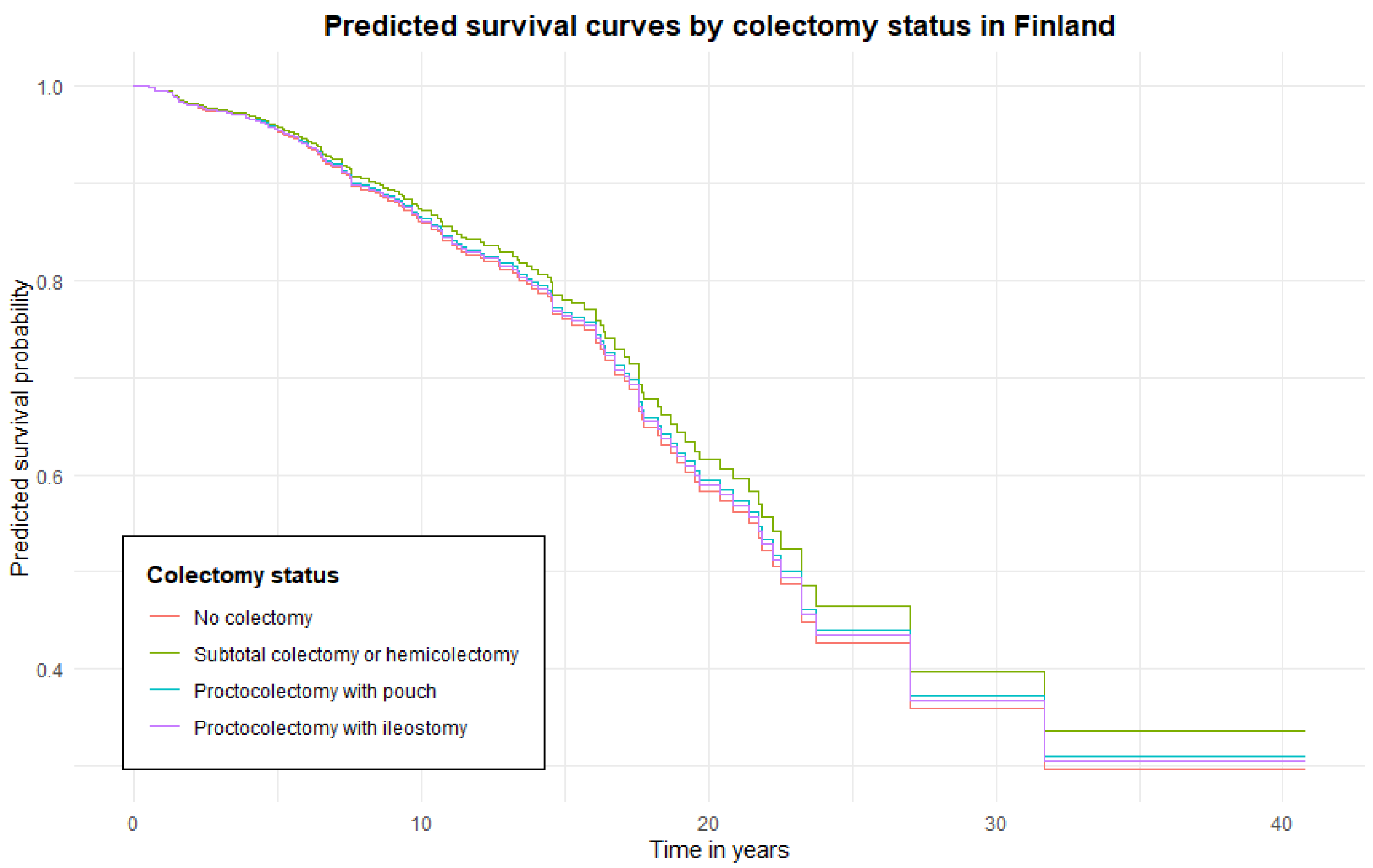
Predicted survival curves by colectomy status in Finland
1.0
0.8
0.6
0.4
Predicted survival probability
0
10
20
30
40
Time in years
Colectomy status
No colectomy
Subtotal colectomy or hemicolectomy
Proctocolectomy with pouch
Proctocolectomy with ileostomy

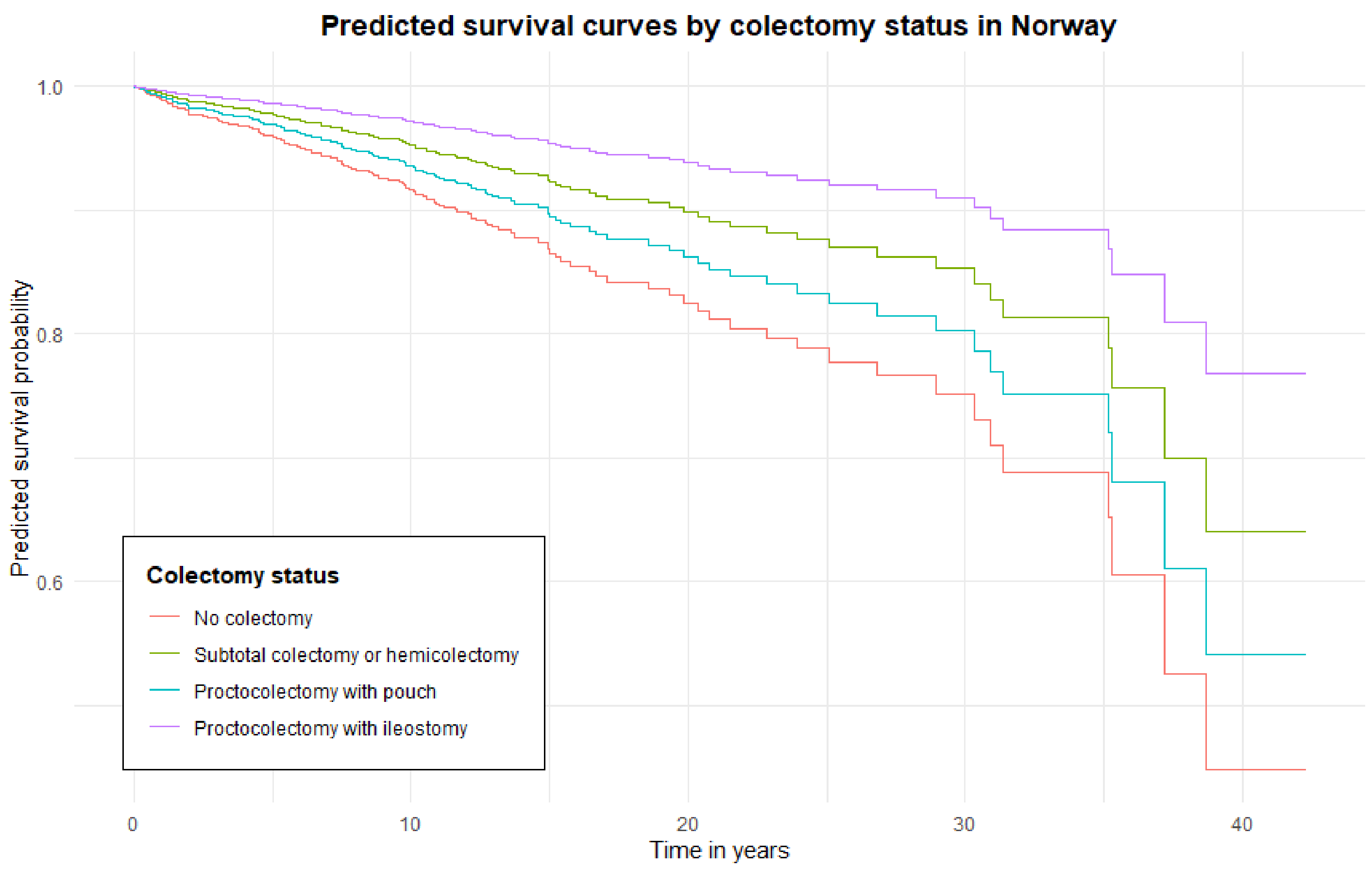

Predicted survival curves by colectomy status in Norway
Predicted survival probability
1.0
0.8
0.6
0
10
20
30
40
Time in years
Colectomy status
No colectomy
Subtotal colectomy or hemicolectomy
Proctocolectomy with pouch
Proctocolectomy with ileostomy

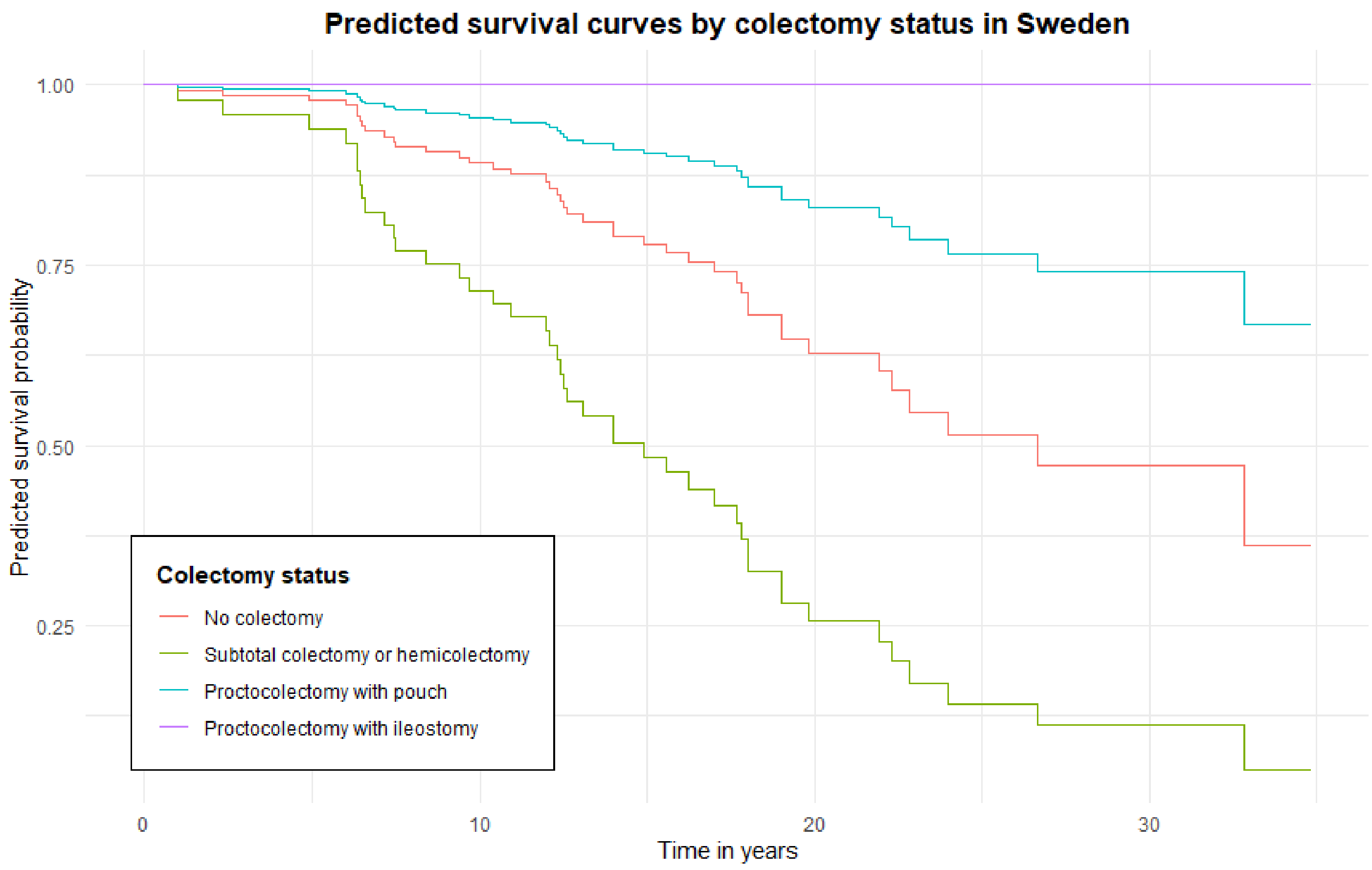
Predicted survival curves by colectomy status in Sweden
1.00
0.75
0.50
0.25
Predicted survival probability
0
10
20
30
Time in years
Colectomy status
No colectomy
Subtotal colectomy or hemicolectomy
Proctocolectomy with pouch
Proctocolectomy with ileostomy

Table S2. Multivariable model results for the sensitivity analysis with assigning events within the first year after colectomy to the pre-colectomy period (A) and without censoring events within one year after colectomy (B)

A. Events within the first year after colectomy assigned to the pre-colectomy period		HR (95%CI)
Colectomy	*None*<br>Subtotal colectomy or<br>hemicolectomy*Proctocolectomy with*<br>*pouch*<br>*Proctocolectomy with ileostomy*	Reference<br>0.91 (0.67-1.25)<br>1.09 (0.81-1.48)<br>0.47 (0.28-0.79)
Female		0.79 (0.69-0.92)
Age at diagnosis (per year)		1.02 (1.02-1.03)
PSC type	*Large duct*<br>*Small duct*<br>*Features of AIH*	Reference<br>0.22 (0.11-0.45)<br>1.06 (0.84-1.34)
IBD	*No IBD*<br>*Ulcerative colitis*<br>*Crohn's disease*<br>*IBD-unspecified*	Reference<br>1.02 (0.86-1.20)<br>0.80 (0.63-1.01)<br>0.95 (0.63-1.43)
Country	*the Netherlands*<br>*Finland*	Reference<br>0.58 (0.42-0.81)

	Norway<br>*Sweden*	0.78 (0.66-0.94)<br>0.36 (0.30-0.44)
Transplant centre patient		1.82 (1.58-2.09)
B. Without censoring events within one year after colectomy		HR (95%CI)
Colectomy	*None*<br>Subtotal colectomy or hemicolectomy<br>*Proctocolectomy with pouch*<br>*Proctocolectomy with ileostomy*	Reference<br>0.94 (0.69-1.27)<br>1.09 (0.81-1.49)<br>0.50 (0.30-0.83)
Female		0.79 (0.68-0.92)
Age at diagnosis (per year)		1.02 (1.02-1.03)
PSC type	*Large duct*<br>*Small duct*<br>*Features of AIH*	Reference<br>0.22 (0.11-0.45)<br>1.06 (0.84-1.34)
IBD	*No IBD*<br>*Ulcerative colitis*<br>*Crohn's disease*<br>*IBD-unspecified*	Reference<br>1.02 (0.86-1.20)<br>0.80 (0.63-1.02)<br>0.95 (0.63-1.43)
Country	*the Netherlands*<br>*Finland*	Reference<br>0.58 (0.42-0.80)

	Norway<br>*Sweden*	0.78 (0.65-0.93)<br>0.36 (0.30-0.44)
Transplant centre patient		1.81 (1.58-2.09)

Table S3. Multivariable model results for transplant-free survival defined as time up to liver transplantation of PSC-related death (excluding colorectal carcinoma) for only participants with a diagnosis of both PSC and IBD.

A. Liver transplantation (excluding HB malignancy) or death due to liver failure		HR (95%CI)
Colectomy	*None*	Reference
	Subtotal colectomy or hemicolectomy	0.76 (0.53-1.09)
	Proctocolectomy with pouch	0.55 (0.24-1.27)
	Proctocolectomy with ileostomy	0.24 (0.09-0.61)
Female		0.85 (0.71-1.01)
Age at diagnosis (per year)		1.02 (1.02-1.03)
PSC type	*Large duct*	Reference
	Small duct	0.26 (0.10-0.69)
	Features of AIH	1.04 (0.78-1.38)
IBD	*No IBD*	Reference
	Ulcerative colitis	0.56 (0.26-1.24)
	Crohn's disease	0.44 (0.20-0.99)
	IBD-unspecified	0.52 (0.22-1.24)
Country	*the Netherlands*	Reference
	Finland	0.50 (0.35-0.73)
	Norway	0.75 (0.61-0.92)
	Sweden	0.38 (0.30-0.47)

Transplant centre patient		1.64 (1.40-1.93)

Table S4. Multivariable model results for the secondary endpoints liver transplantation (excluding hepatobiliary malignancy) or death due to liver failure (A) and occurrence of hepatobiliary malignancy (B)

A. Liver transplantation (excluding HB malignancy) or death due to liver failure		HR (95%CI)
Colectomy	*None*	Reference
	Subtotal colectomy or hemicolectomy	0.65 (0.43-0.97)
	Proctocolectomy with pouch	0.75 (0.50-1.11)
	Proctocolectomy with ileostomy	0.24 (0.10-0.53)
Female		0.81 (0.69-0.96)
Age at diagnosis (per year)		1.02 (1.01-1.02)
PSC type	*Large duct*	Reference
	Small duct	0.18 (0.08-0.45)
	Features of AIH	1.03 (0.80-1.34)
IBD	*No IBD*	Reference
	Ulcerative colitis	1.07 (0.89-1.29)
	Crohn's disease	0.78 (0.60-1.02)
	IBD-unspecified	0.98 (0.62-1.67)
Country	*the Netherlands*	Reference
	Finland	0.71 (0.51-1.00)
	Norway	0.74 (0.60-0.91)
	Sweden	0.33 (0.2-0.41)

Transplant centre patient		2.00 (1.71-2.36)
B. Occurrence of HB malignancy		HR (95%CI)
Colectomy	*None*	Reference
	Subtotal colectomy or hemicolectomy	1.26 (0.75-2.13)
	Proctocolectomy with pouch	1.62 (0.96-2.75)
	Proctocolectomy with ileostomy	1.17 (0.60-2.29)
Female		0.74 (0.56-1.00)
Age at diagnosis (per year)		1.04 (1.03-1.05)
PSC type	*Large duct*	Reference
	Small duct	0.48 (0.18-1.31)
	Features of AIH	0.96 (0.57-1.63)
IBD	*No IBD*	Reference
	Ulcerative colitis	1.01 (0.73-1.42)
	Crohn's disease	0.91 (0.57-1.47)
	IBD-unspecified	1.22 (0.57-2.59)
Country	*the Netherlands*	Reference
	Finland	0.00 (0.00-N.A.)
	Norway	1.00 (0.73-1.38)
	Sweden	1.11 (0.74-1.69)

Transplant centre patient		2.02 (1.51-2.70)

Table S5. Colectomy indication stratified by colectomy type

	Hemicolectomy (N=39)	Subtotal colectomy (N=93)	Proctocolectomy with ileal-anal pouch anastomosis (N=232)	Proctocolectomy with end-ileostomy (N=80)
Active disease	11 (28%)	48 (52%)	116 (50%)	48 (50%)
Active disease and neoplasia	0 (0%)	2 (2%)	9 (4%)	1 (1%)
Dysplasia or carcinoma	21 (54%)	38 (41%)	79 (34%)	29 (36%)
Other	6 (15%)	2 (2%)	3 (1%)	0 (0%)
Unknown	1 (3%)	3 (3%)	25 (11%)	8 (10%)
Colectomy indication stratified by colectomy type.				

Table S6. Multivariable model results for indication of colectomy for the endpoint liver transplantation or PSC-related death (excluding colorectal carcinoma) including only time at risk after any type of colectomy

A. Liver transplantation or PSC-related death (excluding colorectal carcinoma)		HR (95%CI)
Colectomy indication	*Active disease*	Reference
	Active disease and neoplasia	1.42 (0.49-4.11)
	Dysplasia or carcinoma	0.74 (0.46-1.19)
	Other	1.77 (0.68-4.64)
	Unknown	1.10 (0.54-2.26)
Colectomy	*None*	N.A.
	Subtotal colectomy or hemicolectomy	2.07 (0.77-5.54)
	Proctocolectomy with pouch	2.01 (1.04-3.88)
	Proctocolectomy with ileostomy	Reference
Female		1.27 (0.81-2.01)
Age at diagnosis (per year)		1.01 (0.99-1.02)
PSC type	*Large duct*	Reference
	Small duct	0.32 (0.04-2.38)
	Features of AIH	1.44 (0.68-3.06)
IBD	*No IBD*	Reference
	Ulcerative colitis	0.75 (0.30-1.87)

	Crohn's disease<br>*IBD-unspecified*	0.50 (0.16-1.59)<br>0.56 (0.11-2.81)
Country	*the Netherlands*<br>*Finland*<br>*Norway*<br>*Sweden*	Reference<br>0.39 (0.13-1.13)<br>0.57 (0.34-0.96)<br>0.56 (0.27-1.17)
Transplant centre patient		1.51 (0.95-2.40)

Supplementary references

1. van Munster KN, Mol B, Goet JC, et al. Disease burden in primary sclerosing cholangitis in the Netherlands: A long-term follow-up study. *Liver Int* 2023; **43**(3): 639-48.
2. Boonstra K, Weersma RK, van Erpecum KJ, et al. Population-based epidemiology, malignancy risk, and outcome of primary sclerosing cholangitis. *Hepatology* 2013; **58**(6): 2045-55.
3. Villard C, Friis-Liby I, Rorsman F, et al. Prospective surveillance for cholangiocarcinoma in unselected individuals with primary sclerosing cholangitis. *J Hepatol* 2023; **78**(3): 604-13.
